# Intracellular Crosstalk of the Gasotransmitter Trio (NO, CO, H_2_S) in Cardiovascular Health and Disease: From Molecular Signaling to Precision Gas Medicine

**DOI:** 10.3390/ijms27146248

**Published:** 2026-07-14

**Authors:** Tzong-Shyuan Lee

**Affiliations:** Graduate Institute and Department of Physiology, College of Medicine, National Taiwan University, Taipei 10051, Taiwan; ntutslee@ntu.edu.tw

**Keywords:** nitric oxide, carbon monoxide, hydrogen sulfide, ferroptosis, autophagy, mitophagy, redox signaling, nanomedicine, smart delivery systems, precision gas medicine

## Abstract

Nitric oxide (NO), carbon monoxide (CO), and hydrogen sulfide (H_2_S) were once regarded solely as toxic environmental gases. However, accumulating evidence over the past several decades has established them as the three principal endogenous gasotransmitters that regulate a wide spectrum of physiological and pathological processes. Unlike conventional signaling molecules, gasotransmitters diffuse freely across biological membranes and exert potent biological effects through receptor-independent mechanisms, including redox-sensitive post-translational modifications and modulation of heme-containing proteins. Although the individual functions of NO, CO, and H_2_S have been extensively reviewed, emerging studies indicate that these gaseous mediators rarely operate in isolation. Instead, they form a highly integrated signaling network characterized by direct chemical interactions, reciprocal enzymatic regulation, and convergence upon common downstream pathways. In this mini-review, we propose the concept of a “Gasotransmitter Trio Network,” emphasizing the molecular crosstalk among NO, CO, and H_2_S as a fundamental determinant of cellular homeostasis. We first summarize the biosynthetic pathways and major signaling mechanisms of the gasotransmitter trio, including S-nitrosylation, persulfidation, and heme-dependent regulation. We then discuss recent advances revealing how interactions among these gases generate novel bioactive intermediates and coordinate redox signaling. Particular attention is given to the emerging roles of gasotransmitters in regulating ferroptosis, autophagy, and mitophagy by modulating iron metabolism, lipid peroxidation, mitochondrial quality control, and antioxidant defense systems. These findings support a unified framework in which gasotransmitters function as master regulators of cellular fate under conditions of physiological and pathological stress. Finally, we highlight recent progress in stimuli-responsive donors, CO-releasing molecules (CORMs), NO-releasing materials (NORMs), H_2_S donors, and advanced nanoplatforms that enable spatiotemporally controlled gas delivery. We propose that future therapeutic strategies will increasingly rely on programmable multi-gas systems that recapitulate endogenous gasotransmitter networks. Collectively, this review provides a systems-level perspective on gasotransmitter biology and outlines emerging opportunities for the development of precision gas medicine in cardiovascular, neurodegenerative, inflammatory, metabolic, and malignant diseases.

## 1. Introduction

### 1.1. From Environmental Toxins to Endogenous Signaling Molecules: A Paradigm Shift

For much of modern scientific history, nitric oxide (NO), carbon monoxide (CO), and hydrogen sulfide (H_2_S) were primarily regarded as hazardous environmental pollutants and toxic gases. NO was associated with atmospheric pollution and oxidative injury, CO was notorious for its high affinity toward hemoglobin and its potentially lethal effects on oxygen transport, whereas H_2_S was historically recognized as a poisonous gas responsible for industrial and environmental intoxication [1,2,3]. Consequently, these molecules were traditionally viewed as harmful byproducts with limited physiological significance.

This perception changed dramatically during the late twentieth century through a series of landmark discoveries. The identification of NO as the endothelium-derived relaxing factor (EDRF) by Furchgott and colleagues revolutionized cardiovascular biology and established NO as a fundamental regulator of vascular homeostasis [4,5,6]. The discovery of NO as an endogenous signaling molecule revolutionized cardiovascular biology and transformed our understanding of vascular regulation, culminating in the 1998 Nobel Prize in Physiology or Medicine being awarded to Furchgott, Ignarro, and Murad for their discoveries concerning NO as a signaling molecule in the cardiovascular system [4,5,6,7]. Subsequently, CO was found to be generated endogenously during heme degradation by heme oxygenase (HO) enzymes and to exert anti-inflammatory, anti-apoptotic, and cytoprotective effects through intracellular signaling mechanisms [8,9,10]. Shortly thereafter, H_2_S emerged as the third recognized gasotransmitter when mammalian tissues were shown to synthesize H_2_S enzymatically and utilize it to regulate neuronal, cardiovascular, and metabolic functions [11,12,13].

These discoveries collectively transformed the conceptual framework of gaseous molecules in biology. Rather than serving solely as environmental toxins, NO, CO, and H_2_S are now recognized as endogenous signaling molecules that participate in the regulation of virtually every major physiological system [14,15,16]. Similarly to classical neurotransmitters and hormones, gasotransmitters transmit biological information; however, they possess several unique characteristics that distinguish them from conventional signaling mediators.

### 1.2. Physiological Importance of the Gasotransmitter Trio

One of the defining features of gasotransmitters is their ability to diffuse freely across biological membranes without requiring membrane-bound receptors or transport systems [14,17]. This receptor-independent mode of signaling enables rapid communication between intracellular compartments and neighboring cells, thereby facilitating immediate adaptation to environmental and metabolic perturbations. Furthermore, gasotransmitters exert potent biological effects at extremely low concentrations, often in the nanomolar to micromolar range, highlighting the exquisite sensitivity of cellular sensing mechanisms [15,18].

Accumulating evidence indicates that NO, CO, and H_2_S are indispensable regulators of cellular homeostasis. NO controls vascular tone, platelet aggregation, neurotransmission, mitochondrial respiration, and innate immune responses [19,20,21]. CO functions as an important modulator of inflammation, oxidative stress, mitochondrial metabolism, and cellular adaptation to injury through interactions with heme-containing proteins [9,22,23]. Meanwhile, H_2_S participates in antioxidant defense, energy metabolism, angiogenesis, autophagy, and stress adaptation, thereby contributing to tissue protection under physiological and pathological conditions [24,25,26,27]. Importantly, the biological actions of gasotransmitters are highly concentration-dependent. Physiological concentrations generally confer cytoprotective effects, whereas excessive production can contribute to pathological processes. For example, overproduction of NO may induce nitrosative stress via peroxynitrite formation, excessive CO exposure may cause tissue hypoxia via hemoglobin binding, and elevated H_2_S concentrations may inhibit mitochondrial respiration by targeting cytochrome c oxidase [28,29,30]. Therefore, the physiological outcome of gasotransmitter signaling depends on a finely regulated balance involving concentration, spatial distribution, and temporal dynamics.

**Figure 1:**
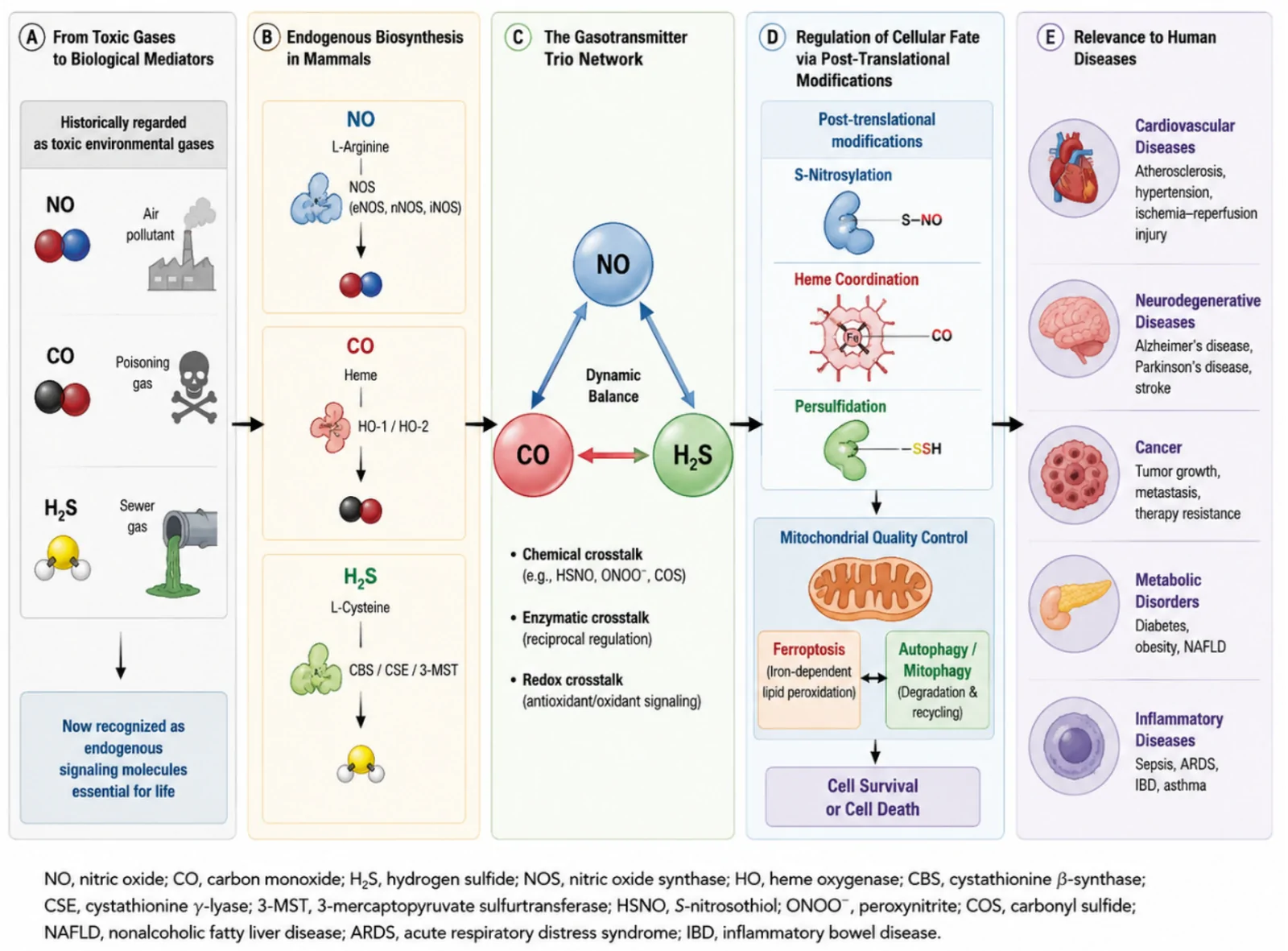
**Evolution of the Gasotransmitter Trio from Toxic Gases to Master Regulators of Cellular Fate.** Nitric oxide (NO), carbon monoxide (CO), and hydrogen sulfide (H_2_S) were historically regarded as toxic environmental gases. Subsequent discoveries demonstrated that these molecules are enzymatically synthesized in mammalian tissues through nitric oxide synthases (NOS), heme oxygenases (HO-1/HO-2), and sulfur-metabolizing enzymes, including cystathionine β-synthase (CBS), cystathionine γ-lyase (CSE), and 3-mercaptopyruvate sulfurtransferase (3-MST). Increasing evidence indicates that NO, CO, and H_2_S form an integrated gasotransmitter network through direct chemical interactions, reciprocal enzymatic regulation, and shared redox signaling pathways. Their biological effects are mediated primarily through post-translational modifications, including S-nitrosylation, heme-dependent coordination, and persulfidation, which collectively regulate mitochondrial quality control, ferroptosis, autophagy, and mitophagy. Dysregulation of these pathways contributes to the pathogenesis of cardiovascular, neurodegenerative, metabolic, inflammatory, and malignant diseases.

Recent advances in redox biology have further revealed that gasotransmitters act not merely as diffusible molecules but also as regulators of post-translational modifications (PTMs). Through mechanisms such as S-nitrosylation, persulfidation, and heme-dependent signaling, these gases reshape protein function, intracellular signaling networks, and gene expression programs, thereby exerting profound influences on cellular fate decisions [31,32,33,34]. Collectively, these findings have transformed the traditional perception of NO, CO, and H_2_S from isolated gaseous mediators into components of a highly coordinated signaling system. As illustrated in Figure 1, the gasotransmitter trio forms an integrated network that links redox regulation, mitochondrial quality control, and cell fate determination, thereby influencing the development and progression of numerous human diseases.

### 1.3. Scope and Novel Perspective of This Review

Although numerous reviews have independently summarized the biological functions of NO, CO, or H_2_S, these approaches often overlook the fact that gasotransmitters rarely function in isolation. Emerging evidence indicates that the three gases form a highly integrated signaling network characterized by direct chemical interactions, reciprocal enzymatic regulation, and convergence on shared downstream pathways [35,36,37,38]. This concept challenges the traditional reductionist view of individual gasotransmitters and instead supports the existence of a dynamic “gasotransmitter trio network” that collectively governs cellular adaptation and survival. It should be noted that the scope of this review is centered on intracellular molecular crosstalk, namely, the direct chemical interactions, reciprocal enzymatic regulation, and convergent downstream signaling among NO, CO, and H_2_S generated within the same cell, rather than on intercellular gasotransmitter exchange between distinct cellular sources (e.g., endothelium-to-smooth-muscle or paracrine transmission), which represents a separate and equally important area of investigation beyond the present scope. In parallel, increasing attention has been paid to the role of gasotransmitters in regulating emerging forms of programmed cell death and quality-control mechanisms. Ferroptosis and autophagy have recently been recognized as critical determinants of tissue injury, aging, neurodegeneration, cardiovascular diseases, metabolic disorders, and cancer progression [39,40,41,42]. Accumulating studies suggest that NO, CO, and H_2_S exert profound influences on iron metabolism, lipid peroxidation, mitochondrial quality control, and autophagic flux, thereby positioning these gases as key regulators of cellular fate [43,44,45,46].

Finally, despite their remarkable therapeutic potential, the clinical translation of gasotransmitter-based interventions remains challenging due to short biological half-lives, narrow therapeutic windows, and systemic toxicities [10,47,48]. Recent developments in stimuli-responsive donors, nanotechnology, and smart biomaterials have created unprecedented opportunities for achieving precise spatiotemporal control of gas delivery [48,49,50]. This gap is evident when the trio-network framework is contrasted with the substantial single-gas review literature. Landmark reviews have comprehensively addressed the cardiovascular roles of NO [19], CO [9], and H_2_S [27] individually, yet each necessarily treats the other two gasotransmitters as background rather than as co-equal, mechanistically interlocked regulators. A single-gas framework cannot readily explain several recurring observations in the cardiovascular literature: paradoxical or context-dependent effects of a given gas (e.g., cardioprotective at physiological concentrations but injurious at pathological ones, Section 3.2); the compensatory upregulation of one gasotransmitter pathway when another is pharmacologically inhibited or genetically deleted; and the disappointing translational record of single-gas donor trials, which have repeatedly shown efficacy in preclinical models but limited or inconsistent benefit in patients. The trio-network perspective adopted in this review addresses these gaps by treating NO, CO, and H_2_S as a single interdependent signaling system that shares chemical intermediates (e.g., HNO, SSNO^−^), reciprocally regulates its own biosynthetic enzymes (CBS, CSE, HO, NOS; Section 3.2), and converges on common downstream effectors, including Nrf2, the sGC-cGMP axis, and mitochondrial quality-control pathways (Section 4), that no single-gas account can fully capture. Therefore, this review focuses on three emerging themes: (i) molecular crosstalk among NO, CO, and H_2_S; (ii) regulation of ferroptosis and autophagy by the gasotransmitter trio; and (iii) next-generation smart delivery systems designed to translate gasotransmitter biology into clinical therapeutics. By integrating these perspectives, we propose a systems-level framework in which the gasotransmitter trio functions as a coordinated regulatory network that determines cellular survival, adaptation, and disease progression.

## 2. Biosynthetic Pathways and Post-Translational Modifications

### 2.1. Enzymatic Production Machinery of the Gasotransmitter Trio

The biological activities of NO, CO, and H_2_S are critically dependent upon tightly regulated enzymatic systems that govern their synthesis, degradation, and spatial distribution. Unlike classical hormones that are stored in vesicles and released upon stimulation, gasotransmitters are synthesized on demand and rapidly diffuse across cellular membranes to exert local and systemic effects [14].

NO is generated from L-arginine by NO synthase (NOS) enzymes, including endothelial NOS (eNOS), neuronal NOS (nNOS), and inducible NOS (iNOS) [19]. These isoforms exhibit distinct patterns of expression and regulation. eNOS-derived NO plays a pivotal role in vascular homeostasis by promoting vasodilation, inhibiting platelet aggregation, and suppressing leukocyte adhesion. In contrast, nNOS primarily regulates neurotransmission and synaptic plasticity, whereas iNOS is induced during inflammation and produces substantially larger quantities of NO as part of the host defense response [21,51]. The activity of NOS enzymes is tightly controlled by intracellular calcium, phosphorylation events, protein–protein interactions, and cofactor availability, particularly tetrahydrobiopterin (BH_4_). Disruption of these regulatory mechanisms may result in NOS uncoupling, leading to superoxide generation rather than NO production, thereby contributing to oxidative stress and vascular dysfunction [52].

CO is primarily generated during heme catabolism catalyzed by heme oxygenase (HO) enzymes [53]. HO-1 is an inducible stress-responsive isoform that is upregulated by oxidative stress, hypoxia, inflammation, and electrophilic stimuli, whereas HO-2 is constitutively expressed in various tissues, including the brain and vasculature [54]. During heme degradation, HO enzymes generate equimolar amounts of biliverdin, ferrous iron (Fe^2+^), and CO. Although historically considered merely a metabolic byproduct, endogenous CO is now recognized as an important signaling molecule that regulates inflammation, mitochondrial metabolism, apoptosis, and vascular function [10].

H_2_S is synthesized predominantly through three enzymatic pathways involving cystathionine β-synthase (CBS), cystathionine γ-lyase (CSE), and 3-mercaptopyruvate sulfurtransferase (3-MST) [55]. CBS is highly expressed in the central nervous system, whereas CSE predominates in the cardiovascular system and liver. In contrast, 3-MST is localized primarily within mitochondria and contributes significantly to intracellular sulfur metabolism [56]. Emerging evidence indicates that H_2_S production is dynamically regulated by nutrient availability, redox status, and cellular stress responses, linking sulfur metabolism to energy homeostasis and adaptive signaling [25].

**Figure 2:**
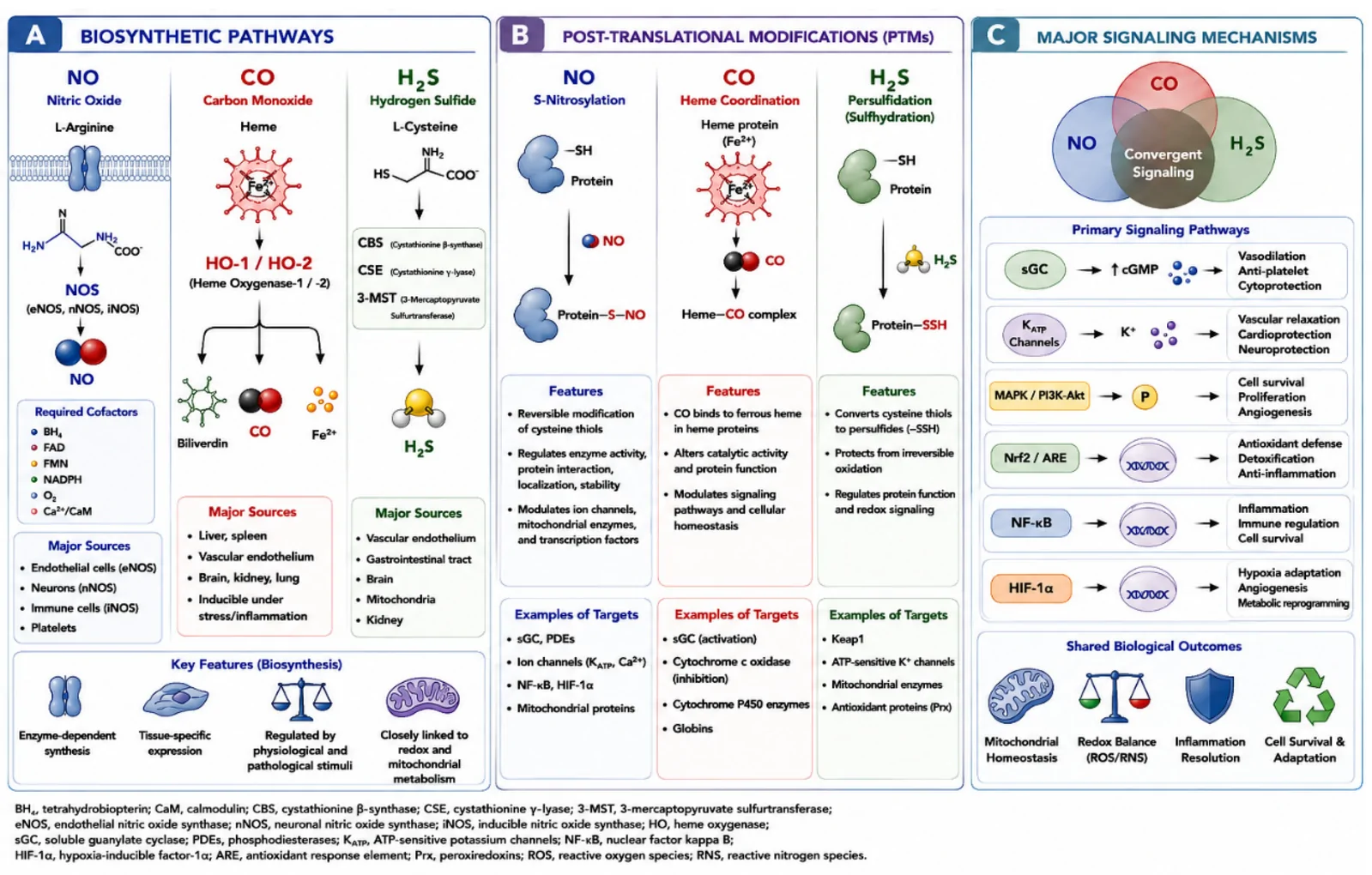
**Biosynthetic pathways, post-translational modifications, and major signaling mechanisms of the gasotransmitter trio.** Nitric oxide (NO), carbon monoxide (CO), and hydrogen sulfide (H_2_S) are synthesized through distinct enzymatic pathways but ultimately converge on shared signaling networks that regulate cellular homeostasis. (**A**) Biosynthetic pathways. NO is generated from L-arginine by nitric oxide synthase (NOS) isoforms, including endothelial NOS (eNOS), neuronal NOS (nNOS), and inducible NOS (iNOS), in the presence of cofactors such as tetrahydrobiopterin (BH_4_), flavin adenine dinucleotide (FAD), flavin mononucleotide (FMN), and NADPH. CO is produced during heme degradation by heme oxygenase-1 (HO-1) and heme oxygenase-2 (HO-2), generating CO, biliverdin, and ferrous iron (Fe^2+^). H_2_S is synthesized primarily from sulfur-containing amino acids by cystathionine β-synthase (CBS), cystathionine γ-lyase (CSE), and 3-mercaptopyruvate sulfurtransferase (3-MST), with tissue-specific expression patterns. (**B**) Gasotransmitter-mediated post-translational modifications (PTMs). NO regulates protein function through reversible S-nitrosylation of reactive cysteine residues, thereby influencing enzymatic activity, protein stability, intracellular trafficking, and signal transduction. CO exerts its biological effects primarily through heme coordination, in which binding to ferrous heme centers modulates the activity of heme-containing proteins, including soluble guanylate cyclase (sGC), cytochrome c oxidase, cytochrome P450 enzymes, and globins. H_2_S mediates protein persulfidation (sulfhydration), converting cysteine thiols (-SH) into persulfides (-SSH), thereby protecting proteins from irreversible oxidation and regulating diverse cellular signaling pathways. (**C**) Major signaling mechanisms and downstream targets. Despite their distinct chemical properties, the three gasotransmitters converge on several shared signaling pathways. Direct targets include soluble guanylate cyclase (sGC), ATP-sensitive potassium (KATP) channels, calcium-sensitive ion channels, and multiple heme-containing proteins. These interactions modulate cyclic guanosine monophosphate (cGMP) signaling, membrane excitability, mitochondrial respiration, and cellular metabolism. Furthermore, NO, CO, and H_2_S regulate redox-sensitive pathways including Nrf2/ARE, NF-κB, and HIF-1α signaling, thereby influencing antioxidant defense, inflammatory responses, hypoxic adaptation, and mitochondrial quality control. Through coordinated regulation of mitochondrial biogenesis, electron transport chain activity, and reactive oxygen/nitrogen species (ROS/RNS) homeostasis, the gasotransmitter trio serves as a central regulator of cellular adaptation and survival under physiological and pathological conditions. ARE, antioxidant response element; BH_4_, tetrahydrobiopterin; CBS, cystathionine β-synthase; CSE, cystathionine γ-lyase; FMN, flavin mononucleotide; FAD, flavin adenine dinucleotide; HIF-1α, hypoxia-inducible factor-1α; HO, heme oxygenase; KATP, ATP-sensitive potassium channel; NF-κB, nuclear factor kappa B; Nrf2, nuclear factor erythroid 2-related factor 2; NOS, nitric oxide synthase; ROS, reactive oxygen species; RNS, reactive nitrogen species; sGC, soluble guanylate cyclase; 3-MST, 3-mercaptopyruvate sulfurtransferase.

Importantly, increasing evidence suggests that the biological effects of gasotransmitters cannot be explained solely by their concentrations. Rather, their signaling specificity arises largely from their ability to induce selective post-translational modifications (PTMs) of target proteins, thereby influencing cellular signaling networks at multiple levels [57]. Collectively, these observations support the concept that gasotransmitter signaling is organized as a hierarchical system encompassing enzymatic biosynthesis, molecular target modification, and downstream signal integration. Figure 2 provides an overview of the biosynthetic pathways, major post-translational modifications, and shared signaling mechanisms through which NO, CO, and H_2_S coordinate cellular responses to physiological and pathological stress.

Collectively, NO, CO, and H_2_S exhibit distinct biosynthetic origins and molecular signaling mechanisms, yet ultimately converge on overlapping pathways that regulate redox homeostasis, mitochondrial function, and cellular adaptation. While NO primarily signals via S-nitrosylation, CO acts via heme-dependent interactions, and H_2_S predominantly mediates protein persulfidation. Despite these differences, the three gasotransmitters share numerous downstream targets and biological functions. To facilitate comparison of their biosynthetic pathways, signaling mechanisms, and physiological actions, the major characteristics of the gasotransmitter trio are summarized in Table 1.

As summarized in Table 1, the unique chemical properties of each gasotransmitter provide signaling specificity, whereas their overlapping biological activities establish the foundation for coordinated cellular regulation. This functional convergence underlies the extensive molecular interactions discussed in the following section.

### 2.2. Beyond Classical Receptors: Gasotransmitter-Dependent Post-Translational Modifications

Unlike conventional signaling molecules that typically activate membrane-bound receptors, gasotransmitters often act through direct chemical modification of proteins. These PTMs serve as molecular switches that rapidly alter protein structure, enzymatic activity, intracellular localization, and protein–protein interactions [31].

#### 2.2.1. S-Nitrosylation: The Signature Modification of NO

Among NO-mediated PTMs, S-nitrosylation is the best characterized and involves the covalent attachment of a NO moiety to reactive cysteine thiols, forming S-nitrosothiols (SNOs) [58]. This reversible modification functions analogously to phosphorylation and has emerged as a fundamental mechanism of redox signaling.

S-nitrosylation regulates a wide spectrum of proteins involved in mitochondrial function, apoptosis, inflammation, and metabolism. One notable example is dynamin-related protein 1 (Drp1), a master regulator of mitochondrial fission. Excessive S-nitrosylation of Drp1 enhances mitochondrial fragmentation and has been implicated in neurodegenerative disorders such as Alzheimer’s disease and Parkinson’s disease [59]. Similarly, S-nitrosylation influences the activity of caspases, NF-κB signaling components, and mitochondrial respiratory complexes, thereby integrating NO signaling with cellular survival pathways [60].

Under physiological conditions, S-nitrosylation contributes to adaptive stress responses and tissue protection. However, excessive nitrosative stress may result in aberrant protein modification and cellular dysfunction, highlighting the dual nature of NO-dependent signaling [61].

#### 2.2.2. Persulfidation: A Protective Redox Mechanism of H_2_S

Persulfidation, also known as sulfhydration, is the principal signaling mechanism of H_2_S [33]. In this process, a cysteine thiol (-SH) is converted into a persulfide group (-SSH), thereby altering the biochemical properties of target proteins. Recent proteomic analyses suggest that persulfidation is one of the most widespread redox-dependent PTMs in mammalian cells and may affect thousands of proteins [62].

Unlike many oxidative modifications that impair protein function, persulfidation frequently exerts protective effects by shielding reactive cysteine residues from irreversible oxidation [63]. This mechanism is particularly important under conditions of oxidative stress. One of the best-characterized examples involves Kelch-like ECH-associated protein 1 (Keap1), a cytoplasmic inhibitor of nuclear factor erythroid 2-related factor 2 (Nrf2). Persulfidation of Keap1 promotes dissociation of the Keap1–Nrf2 complex, enabling Nrf2 nuclear translocation and activation of antioxidant gene expression programs [64]. Through this mechanism, H_2_S enhances cellular resistance to oxidative injury, inflammation, and ferroptosis. Beyond redox regulation, persulfidation also influences mitochondrial bioenergetics, autophagy, endoplasmic reticulum stress responses, and metabolic adaptation, highlighting the broad physiological importance of sulfur-based signaling [65].

#### 2.2.3. Heme Coordination: CO as a Metabolic Regulator

In contrast to NO and H_2_S, the primary signaling mechanism of CO relies on its ability to bind transition metal centers, particularly heme-containing proteins [18]. CO exhibits high affinity for ferrous heme iron, thereby modulating the activity of numerous cellular sensors and enzymes. One major target of CO is cytochrome c oxidase (Complex IV) within the mitochondrial electron transport chain. At low concentrations, transient inhibition of cytochrome c oxidase induces mild mitochondrial stress, activating adaptive signaling pathways, including mitochondrial biogenesis, antioxidant responses, and metabolic reprogramming [66]. This phenomenon resembles mitohormesis, whereby low-level stress enhances cellular resilience.

CO can also interact with soluble guanylate cyclase (sGC), the canonical receptor for NO. Although CO activates sGC less efficiently than NO, it contributes to cGMP-dependent signaling under specific physiological contexts [67]. Moreover, CO-mediated modulation of heme-containing transcription factors and redox-sensitive proteins influences inflammatory responses, angiogenesis, and cell survival [68].

Recent studies suggest that CO functions as a fine-tuner of cellular metabolism rather than a simple signaling molecule. Through selective interactions with mitochondrial and heme-associated proteins, CO coordinates energy production, redox homeostasis, and stress adaptation, thereby contributing to tissue protection during pathological conditions such as ischemia–reperfusion injury and chronic inflammation [69].

Collectively, S-nitrosylation, persulfidation, and heme coordination constitute three major molecular languages through which NO, H_2_S, and CO exert biological effects. These PTMs provide the mechanistic foundation for the extensive crosstalk among the gasotransmitter trio discussed in the following section.

## 3. The Molecular Crosstalk Network Among the Gasotransmitter Trio

**Figure 3:**
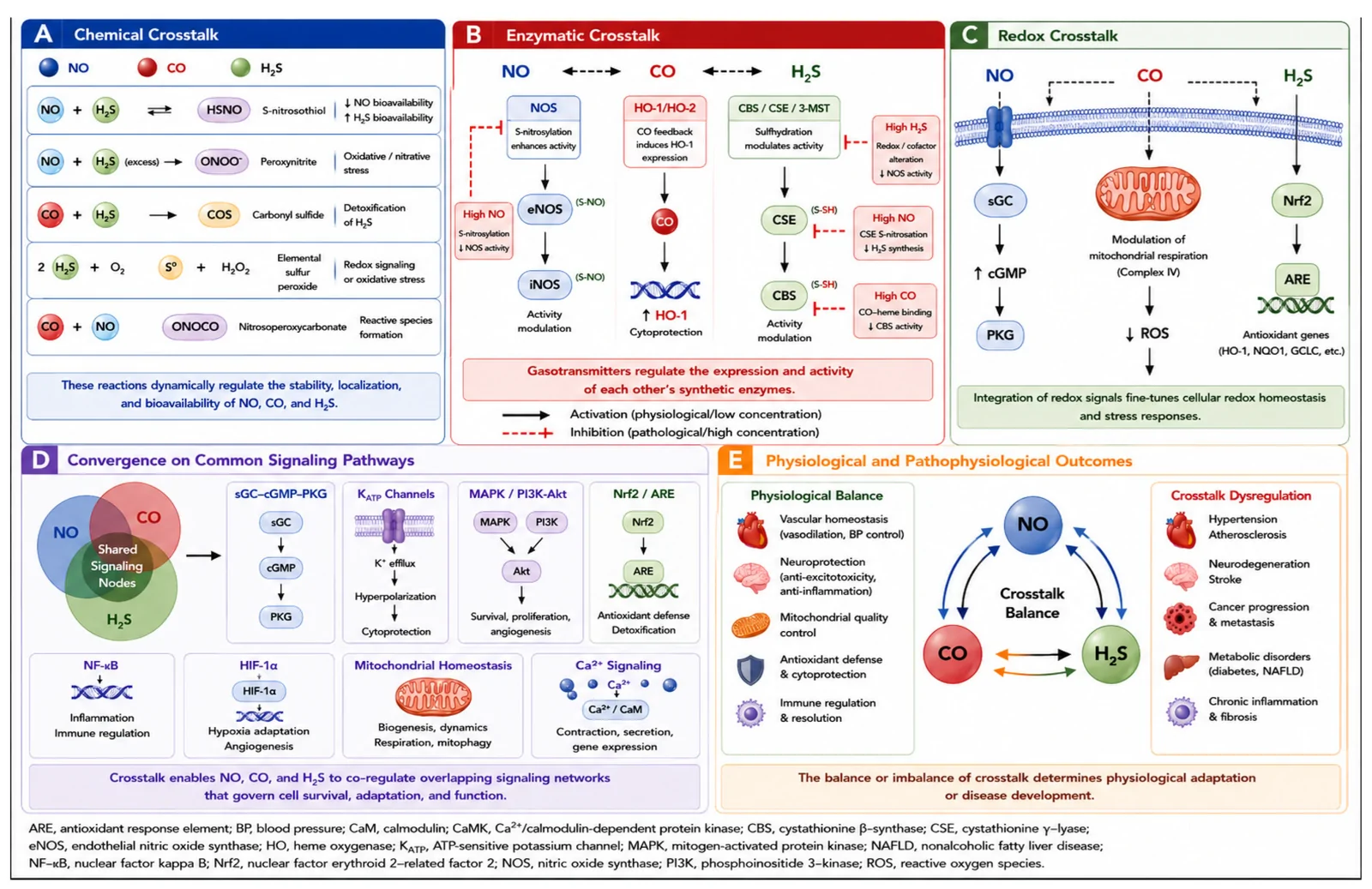
**Molecular crosstalk network among nitric oxide (NO), carbon monoxide (CO), and hydrogen sulfide (H_2_S).** The biological activities of NO, CO, and H_2_S are coordinated through multiple layers of molecular crosstalk, including direct chemical reactions, reciprocal enzymatic regulation, integration of redox signaling, and convergence on shared downstream pathways. (**A**) Chemical crosstalk. NO and H_2_S react to form a variety of reactive sulfur–nitrogen intermediates, including nitrosopersulfide (SSNO^−^), nitroxyl (HNO), and other sulfur–nitrogen hybrid species. These intermediates exhibit distinct biological activities compared with their parent molecules and may serve as prolonged reservoirs of signaling capacity. Interactions among NO, CO, and H_2_S further influence gasotransmitter bioavailability, redox balance, and reactive species formation. (**B**) Enzymatic crosstalk. Gasotransmitter-generating enzymes are regulated by bidirectional feedback mechanisms that depend on local gas concentrations. Under physiological conditions, activating interactions dominate: H_2_S enhances eNOS activity through sulfhydration-dependent mechanisms and the preservation of tetrahydrobiopterin (BH_4_); NO induces HO-1 expression via Nrf2/HIF-1α-dependent pathways to increase CO generation; and H_2_S further promotes HO-1 activation through redox-sensitive signaling. In contrast, excessive accumulation of gasotransmitters triggers negative feedback regulation. High NO levels inhibit NOS activity via S-nitrosylation-mediated enzyme regulation and suppress CSE-dependent H_2_S generation, whereas elevated CO levels inhibit CBS activity through direct heme binding. Excessive H_2_S may also negatively regulate NOS activity through redox- and cofactor-dependent mechanisms. Thus, the CBS/CSE–HO–NOS network functions as a concentration-sensitive regulatory circuit that maintains gasotransmitter homeostasis while preventing pathological overactivation. (**C**) Redox crosstalk. All three gasotransmitters participate in the regulation of cellular redox homeostasis. NO activates soluble guanylate cyclase (sGC) and cGMP signaling, CO modulates mitochondrial respiration through interactions with cytochrome c oxidase (Complex IV), and H_2_S promotes activation of the Nrf2/ARE antioxidant pathway through persulfidation of Keap1. Together, these mechanisms coordinate antioxidant defense, mitochondrial adaptation, and cellular stress responses. (**D**) Convergence on common signaling pathways. Despite their distinct biosynthetic origins, NO, CO, and H_2_S converge on several shared signaling nodes, including the sGC–cGMP–PKG axis, ATP-sensitive potassium (KATP) channels, PI3K/Akt signaling, MAPK pathways, Nrf2-mediated antioxidant responses, NF-κB-dependent inflammatory signaling, HIF-1α-mediated hypoxic adaptation, mitochondrial quality control pathways, and calcium signaling networks. Through these shared pathways, gasotransmitters collectively regulate cell survival, metabolism, angiogenesis, inflammation, and stress adaptation. (**E**) Physiological and pathophysiological outcomes. Balanced gasotransmitter crosstalk supports vascular homeostasis, neuroprotection, mitochondrial fitness, antioxidant defense, and immune regulation. In contrast, dysregulation of the gasotransmitter network contributes to the development of cardiovascular disease, neurodegeneration, metabolic disorders, chronic inflammation, fibrosis, and cancer progression. Thus, cellular adaptation and disease susceptibility are determined not only by the abundance of individual gasotransmitters but also by the integrity of the overall gasotransmitter trio network. ARE, antioxidant response element; BH_4_, tetrahydrobiopterin; CBS, cystathionine β-synthase; CSE, cystathionine γ-lyase; eNOS, endothelial nitric oxide synthase; HIF-1α, hypoxia-inducible factor-1α; HO, heme oxygenase; KATP, ATP-sensitive potassium channel; MAPK, mitogen-activated protein kinase; NF-κB, nuclear factor kappa B; Nrf2, nuclear factor erythroid 2-related factor 2; NOS, nitric oxide synthase; PI3K, phosphoinositide 3-kinase; PKG, protein kinase G; ROS, reactive oxygen species; sGC, soluble guanylate cyclase; SSNO^−^, nitrosopersulfide; HNO, nitroxyl; 3-MST, 3-mercaptopyruvate sulfurtransferase.

Although NO, CO, and H_2_S were initially investigated as independent signaling molecules, accumulating evidence suggests that their biological activities are extensively interconnected. Rather than functioning as isolated mediators, these gasotransmitters form a highly integrated signaling network characterized by direct chemical interactions, reciprocal enzymatic regulation, and convergence upon common downstream signaling pathways [14,16,35,36,37,38]. This dynamic “gasotransmitter trio network” enables cells to coordinate redox homeostasis, mitochondrial adaptation, metabolic flexibility, and stress responses with remarkable precision. Importantly, the biological effects of each gasotransmitter are often influenced by the local abundance of the other two molecules. Consequently, understanding gasotransmitter biology requires a systems-level perspective rather than a reductionist examination of individual signaling pathways. Collectively, current evidence indicates that gasotransmitter signaling is organized as a multilayered regulatory network encompassing chemical interactions, reciprocal enzyme regulation, redox integration, and convergence on common downstream signaling pathways. Rather than acting as isolated mediators, NO, CO, and H_2_S continuously influence each other’s production, bioavailability, and biological activity. The major components of this gasotransmitter crosstalk network are summarized in Figure 3.

The major forms of gasotransmitter crosstalk are summarized in Table 2.

Taken together, these observations support a systems-level model in which NO, CO, and H_2_S function as an integrated signaling network. The biological consequences of this network become particularly evident in the regulation of cellular fate programs, including ferroptosis and autophagy.

### 3.1. Direct Chemical Interactions: Formation of Novel Signaling Intermediates

Among the three gasotransmitters, the interaction between NO and H_2_S has received the greatest attention due to its ability to generate entirely new bioactive signaling species [36,37,70]. Unlike classical signaling cascades that rely on receptor activation, the chemistry of NO and H_2_S allows spontaneous reactions under physiological conditions, producing hybrid sulfur-nitrogen intermediates with distinct biological activities. One of the most extensively studied reaction products is nitrosopersulfide (SSNO^−^), which is formed through interactions between H_2_S-derived persulfides and NO-derived species [36]. SSNO^−^ exhibits significantly greater chemical stability than NO itself and functions as a sustained reservoir of bioactive nitrogen species. Importantly, SSNO^−^ can activate soluble guanylate cyclase (sGC) and promote prolonged vasorelaxation, suggesting that some cardiovascular effects previously attributed solely to NO may actually involve NO/H_2_S hybrid signaling [36,70].

Another important intermediate is nitroxyl (HNO), the one-electron reduced and protonated congener of NO [71]. HNO possesses pharmacological properties distinct from those of NO, including resistance to superoxide scavenging and potent positive inotropic effects in the heart. Experimental studies have demonstrated that HNO improves cardiac contractility while avoiding some of the tolerance mechanisms associated with conventional NO donors [72]. Consequently, HNO has emerged as a promising therapeutic candidate for heart failure and ischemic heart disease.

Beyond SSNO^−^ and HNO, reactions between NO and H_2_S can generate polysulfides and other reactive sulfur-nitrogen species that modulate redox-sensitive proteins and influence cellular signaling networks [33,62]. These findings suggest that the physiological significance of gasotransmitter signaling extends beyond individual molecules and encompasses a broader family of chemically interconnected reactive species.

Compared with NO-H_2_S interactions, direct chemical reactions involving CO are less prominent because CO is relatively inert under physiological conditions. Nevertheless, CO can indirectly influence NO and H_2_S signaling by competing for metal-binding sites and altering the redox environment in which sulfur- and nitrogen-based signaling species are generated [18].

### 3.2. Reciprocal Enzymatic Regulation: A Multi-Layered, Concentration-Dependent Feedback Network

Gasotransmitters regulate each other’s biosynthetic enzymes in a fundamentally concentration-dependent manner: physiological concentrations generally activate the other enzymes, whereas pathological or high concentrations frequently inhibit them. This bidirectional regulation, summarized systematically for all three gases and four enzymes (CBS, CSE, HO, NOS) in Table 3, is described below.

#### 3.2.1. Reciprocal Activation Under Physiological Concentrations

A substantial body of evidence indicates that H_2_S modulates NO production by regulating eNOS activity [35,38,76]. At physiological concentrations, H_2_S promotes eNOS phosphorylation at Ser1177, enhances eNOS dimerization, and increases NO bioavailability [74]. H_2_S also protects tetrahydrobiopterin (BH_4_) from oxidative degradation, preventing eNOS uncoupling [75]. CO similarly activates eNOS and enhances endothelial NO production through PI3K/Akt-dependent mechanisms at low concentrations [77]. Conversely, NO induces HO-1 expression through Nrf2- and HIF-1α-dependent transcriptional pathways, increasing endogenous CO generation [78,79]; this induction is itself concentration-dependent, with higher physiological NO levels producing progressively greater HO-1 promoter activity rather than inhibition [54]. H_2_S likewise induces HO-1 through Nrf2-dependent transcription, while HO-1-derived CO can modulate CBS/CSE activity [80]. Together, these mechanisms establish a physiological-range network in which each gasotransmitter reinforces the production of the other two.

#### 3.2.2. Reciprocal Inhibition Under Pathological/High Concentrations

This activating network reverses at pathological concentrations. Excessive H_2_S suppresses NOS activity by interacting with catalytic cofactors or altering intracellular redox status, producing the biphasic H_2_S–NOS relationship noted above [75]. NO itself is subject to negative-feedback inhibition: at elevated concentrations, S-nitrosylation of cysteine residues at the eNOS dimer interface destabilizes the zinc-tetrathiolate cluster, causing dimer collapse, monomerization, and loss of catalytic activity, a direct product-inhibition loop in which NO limits its own synthesis [52]. CO exerts an analogous inhibitory action on CBS: CO binds reversibly to the enzyme’s prosthetic heme, displacing the endogenous cysteine ligand and completely blocking cystathionine β-synthase activity in a manner competitive with the homocysteine substrate [18]. CSE is subject to a comparable NO-mediated inhibitory mechanism: S-nitrosation of a single solvent-exposed cysteine residue (Cys229 in human CSE) suppresses its H_2_S-generating activity, providing a direct NO-to-H_2_S negative feedback pathway distinct from H_2_S’s own effect on NOS [26]. More broadly, high NO concentrations can reversibly inhibit the insertion of heme into apo-hemoproteins, adding a further layer of concentration-dependent, heme-mediated control over gasotransmitter-synthesizing enzymes [53]. Collectively, these inhibitory mechanisms establish that the same enzymatic nodes activated at physiological gasotransmitter concentrations become sites of negative feedback once those concentrations exceed the physiological range, a bidirectional design that likely protects against excessive, self-amplifying gasotransmitter signaling.

## 4. Dictating Cardiovascular Cell Fate: Roles of Gasotransmitters in Ferroptosis, Autophagy, and Mitophagy

The traditional view of gasotransmitters has largely focused on their roles in vascular regulation, neurotransmission, and inflammatory signaling. However, emerging evidence suggests that NO, CO, and H_2_S exert much broader biological functions by governing fundamental cellular fate decisions. Among these, ferroptosis and autophagy have recently emerged as two highly interconnected processes that determine whether cells adapt to stress or undergo irreversible damage [39,40,41].

**Figure 4:**
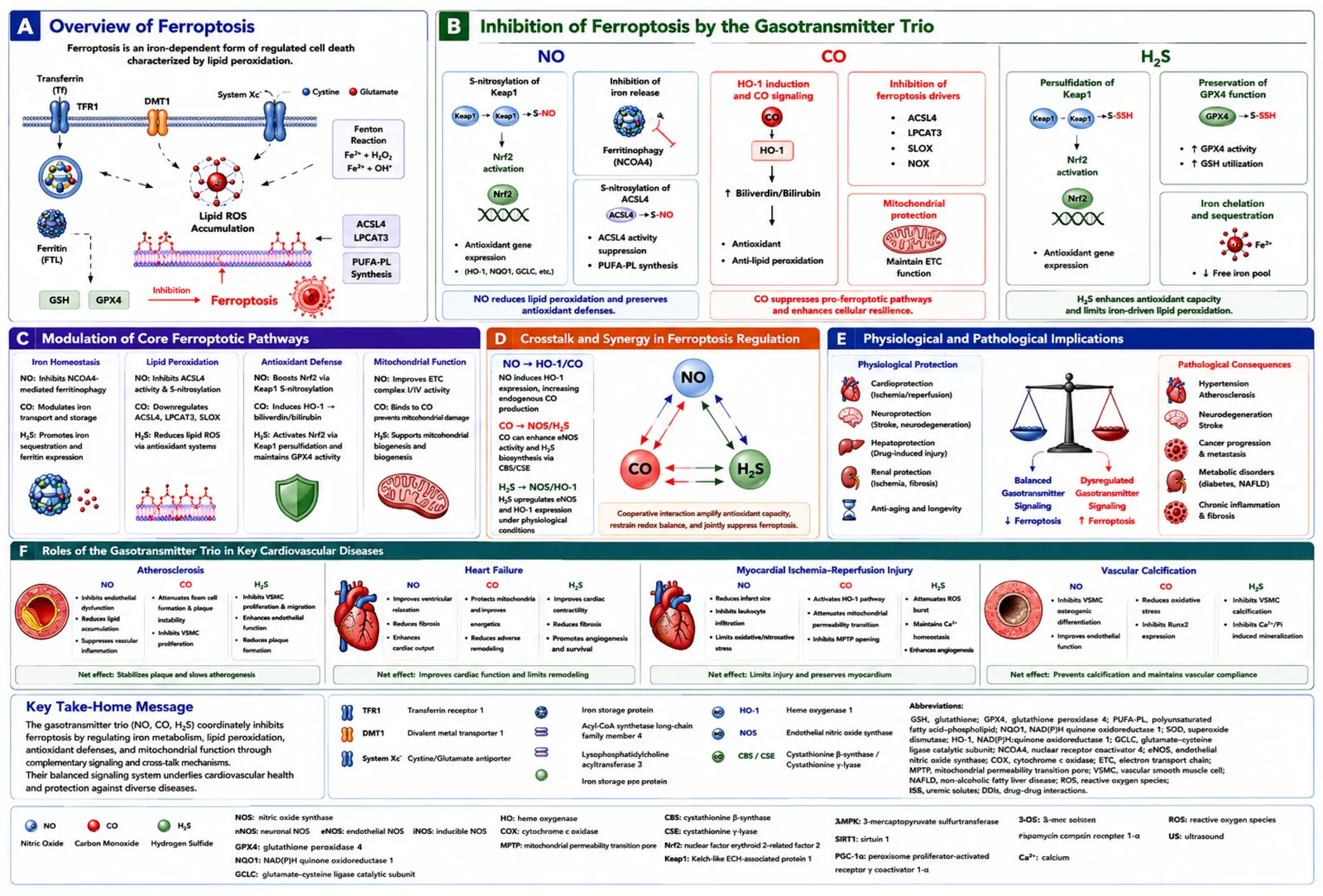
**Regulation of ferroptosis by the gasotransmitter trio (NO, CO, and H_2_S)**. This figure illustrates how the gasotransmitter trio, nitric oxide (NO), carbon monoxide (CO), and hydrogen sulfide (H_2_S), suppresses ferroptosis and protects against major cardiovascular diseases. (**A**) Overview of ferroptosis. Ferroptosis is an iron-dependent, regulated form of cell death driven by excessive lipid peroxidation. Increased intracellular Fe^2+^ (via TfR1/DMT1 uptake and ferritin turnover) generates ROS through the Fenton reaction. ACSL4- and LPCAT3-mediated synthesis of polyunsaturated phospholipids (PUFA-PL) promotes lipid peroxide accumulation, while the GSH-GPX4 axis counteracts this process. (**B**) Inhibition of ferroptosis by the gasotransmitter trio. NO suppresses ferroptosis via S-nitrosylation of Keap1 and ACSL4, activation of Nrf2, inhibition of ferritinophagy, and reduced PUFA-PL synthesis. CO induces HO-1, generates biliverdin/bilirubin, suppresses pro-ferroptotic enzymes (ACSL4, LPCAT3, 5-LOX, NOX), and preserves mitochondrial function. H_2_S inhibits ferroptosis through Keap1 persulfidation, Nrf2 activation, GPX4 preservation, enhanced GSH utilization, and free iron sequestration. (**C**) Modulation of core ferroptotic pathways. The gasotransmitter trio coordinately regulates iron homeostasis, lipid peroxidation, antioxidant defense, and mitochondrial function, thereby reducing iron-driven oxidative stress, membrane lipid oxidation, and mitochondrial dysfunction. (**D**) Crosstalk and synergistic regulation. NO, CO, and H_2_S exhibit extensive bidirectional interactions: NO induces HO-1 and CO production; CO enhances eNOS activity and H_2_S biosynthesis; H_2_S promotes eNOS activation and HO-1 expression. This network amplifies antioxidant defenses and resistance to ferroptosis. (**E**) Physiological and pathological implications. Balanced gasotransmitter signaling suppresses ferroptosis and confers cardioprotection, neuroprotection, and healthy aging. Dysregulated signaling promotes ferroptosis-associated diseases, including atherosclerosis, hypertension, stroke, neurodegeneration, fibrosis, and cancer. (**F**) Roles in major cardiovascular diseases. In atherosclerosis, the trio improves endothelial function, reduces foam-cell formation and oxidative stress, and inhibits plaque progression. In heart failure, it enhances cardiac performance, preserves mitochondrial energetics, and promotes cardiomyocyte survival. During ischemia–reperfusion injury, it limits oxidative damage, mPTP opening, and infarct size. In vascular calcification, it suppresses VSMC osteogenic differentiation and calcium deposition, preserving vascular compliance. ACSL4, acyl-CoA synthetase long-chain family member 4; DMT1, divalent metal transporter 1; GSH, glutathione; GPX4, glutathione peroxidase 4; HO-1, heme oxygenase-1; LPCAT3, lysophosphatidylcholine acyltransferase 3; Nrf2, nuclear factor erythroid 2-related factor 2; NOX, NADPH oxidase; PUFA-PL, polyunsaturated fatty acid-containing phospholipid; ROS, reactive oxygen species; TfR1, transferrin receptor 1.

Ferroptosis is an iron-dependent form of regulated cell death characterized by uncontrolled lipid peroxidation and membrane destruction, whereas autophagy functions primarily as a cytoprotective quality-control mechanism that degrades damaged proteins and organelles [39,44,45,85]. Increasing evidence indicates that gasotransmitters influence both pathways through redox-sensitive post-translational modifications, regulation of mitochondrial homeostasis, and modulation of iron metabolism. Consequently, the gasotransmitter trio may be viewed as a master regulatory network that integrates oxidative stress signals and determines cellular survival under pathological conditions. To better illustrate the multifaceted roles of NO, CO, and H_2_S in ferroptotic regulation, Figure 4 summarizes the major components of the ferroptotic machinery and highlights how the gasotransmitter trio cooperatively modulates iron metabolism, lipid peroxidation, antioxidant defense systems, and mitochondrial integrity. These integrated mechanisms collectively determine ferroptotic susceptibility and influence disease progression across multiple organ systems.

Recent studies have identified ferroptosis and autophagy/mitophagy as two major cellular fate pathways influenced by gasotransmitter signaling. Although the three gases employ distinct molecular mechanisms, they collectively regulate oxidative stress, mitochondrial quality control, and iron metabolism. Their coordinated effects on these pathways are summarized in Table 4.

The integrated regulation of ferroptosis and autophagy highlights a unifying theme in gasotransmitter biology: preservation of mitochondrial integrity and cellular homeostasis. These mechanistic insights also provide a strong rationale for the development of gasotransmitter-based therapeutics and delivery technologies.

### 4.1. Breaking the Code of Ferroptosis: Gasotransmitters as Modulators of Iron-Dependent Cell Death

Ferroptosis is initiated by excessive accumulation of lipid peroxides in cellular membranes and is critically dependent upon intracellular iron availability, glutathione (GSH) depletion, and inactivation of glutathione peroxidase 4 (GPX4) [39,40,41]. Because these processes are tightly linked to cellular redox homeostasis, gasotransmitters are uniquely positioned to influence ferroptotic susceptibility.

#### 4.1.1. NO as a Ferroptosis Suppressor

Among the gasotransmitters, NO exhibits complex and context-dependent effects on ferroptosis. Under physiological conditions, NO can directly terminate lipid peroxidation chain reactions by reacting with lipid peroxyl radicals (LOO•), thereby preventing propagation of oxidative membrane damage [86]. This radical-trapping activity resembles that of classical lipophilic antioxidants and provides an important mechanism by which NO suppresses ferroptotic cell death.

In addition, NO-mediated S-nitrosylation regulates multiple proteins involved in iron metabolism and antioxidant defense [31,58]. Several studies have demonstrated that moderate NO production preserves GPX4 activity, maintains glutathione availability, and suppresses ferroptosis in endothelial cells, neurons, and cardiomyocytes exposed to oxidative stress [86]. Furthermore, NO may modulate iron homeostasis by regulating ferritin expression and iron-responsive signaling pathways, thereby limiting the accumulation of the labile iron pool that drives ferroptotic injury [87].

Nevertheless, excessive NO production may also contribute to ferroptosis under certain pathological conditions. High concentrations of NO can react with superoxide to generate peroxynitrite, thereby causing mitochondrial dysfunction and increased oxidative damage [28]. Thus, the effects of NO on ferroptosis appear to follow a concentration-dependent hormetic pattern.

#### 4.1.2. H_2_S as a Guardian of the GPX4-GSH Axis

H_2_S is increasingly recognized as one of the most potent endogenous inhibitors of ferroptosis [33,43]. A major mechanism involves preservation of intracellular glutathione homeostasis. By stimulating cystine uptake and activating System Xc^−^, H_2_S enhances cellular cysteine availability and promotes glutathione biosynthesis [90].

Moreover, H_2_S-mediated persulfidation directly protects antioxidant proteins from irreversible oxidation [33,63]. Recent studies suggest that H_2_S enhances GPX4 stability and activity, thereby preventing the accumulation of toxic lipid hydroperoxides that trigger ferroptosis [43,91]. Activation of the Keap1-Nrf2 signaling pathway further amplifies this protective response by inducing the expression of antioxidant enzymes, glutathione-synthesis genes, and iron-detoxifying proteins [64].

Another emerging mechanism involves mitochondrial sulfur metabolism. H_2_S supports mitochondrial bioenergetics by donating electrons to the respiratory chain and maintains mitochondrial integrity during oxidative stress [92]. By preserving mitochondrial function and reducing reactive oxygen species (ROS) production, H_2_S indirectly suppresses the oxidative environment that promotes ferroptotic execution.

#### 4.1.3. CO: A Double-Edged Regulator of Ferroptosis

Compared with NO and H_2_S, the role of CO in ferroptosis is considerably more complex. The biological effects of CO are closely linked to the activity of heme oxygenase-1 (HO-1), which simultaneously generates CO, biliverdin, and free iron during heme degradation [8,9,53,54]. At moderate levels, HO-1 activation exerts cytoprotective effects through antioxidant signaling, Nrf2 activation, and suppression of inflammation [9,22,53,69]. These mechanisms indirectly reduce ferroptotic susceptibility and contribute to tissue protection in models of cardiovascular injury [9,22,53,69]. However, excessive HO-1 activation may produce the opposite outcome. Because heme degradation releases ferrous iron, prolonged or excessive HO-1 activity can increase the intracellular labile iron pool and accelerate Fenton chemistry, thereby promoting lipid peroxidation and ferroptosis [88,89]. This dual role has led to the concept that the HO-1/CO system functions as a ferroptotic rheostat, capable of either suppressing or promoting ferroptosis depending on the cellular context and iron-handling capacity.

Collectively, NO, CO, and H_2_S regulate ferroptosis through complementary mechanisms that involve antioxidant defense, iron metabolism, control of lipid peroxidation, and mitochondrial preservation. Their coordinated actions suggest that ferroptotic sensitivity may ultimately depend on the balance of gasotransmitter signaling within individual tissues.

### 4.2. Tuning Autophagy and Mitophagy Flux: The Mitochondrial Quality Control Axis

While ferroptosis determines whether cells succumb to oxidative damage, autophagy represents a critical adaptive mechanism that enables survival under stressful conditions. Autophagy facilitates degradation of damaged proteins, dysfunctional organelles, and toxic macromolecular aggregates, thereby maintaining intracellular homeostasis [44,45].

**Figure 5:**
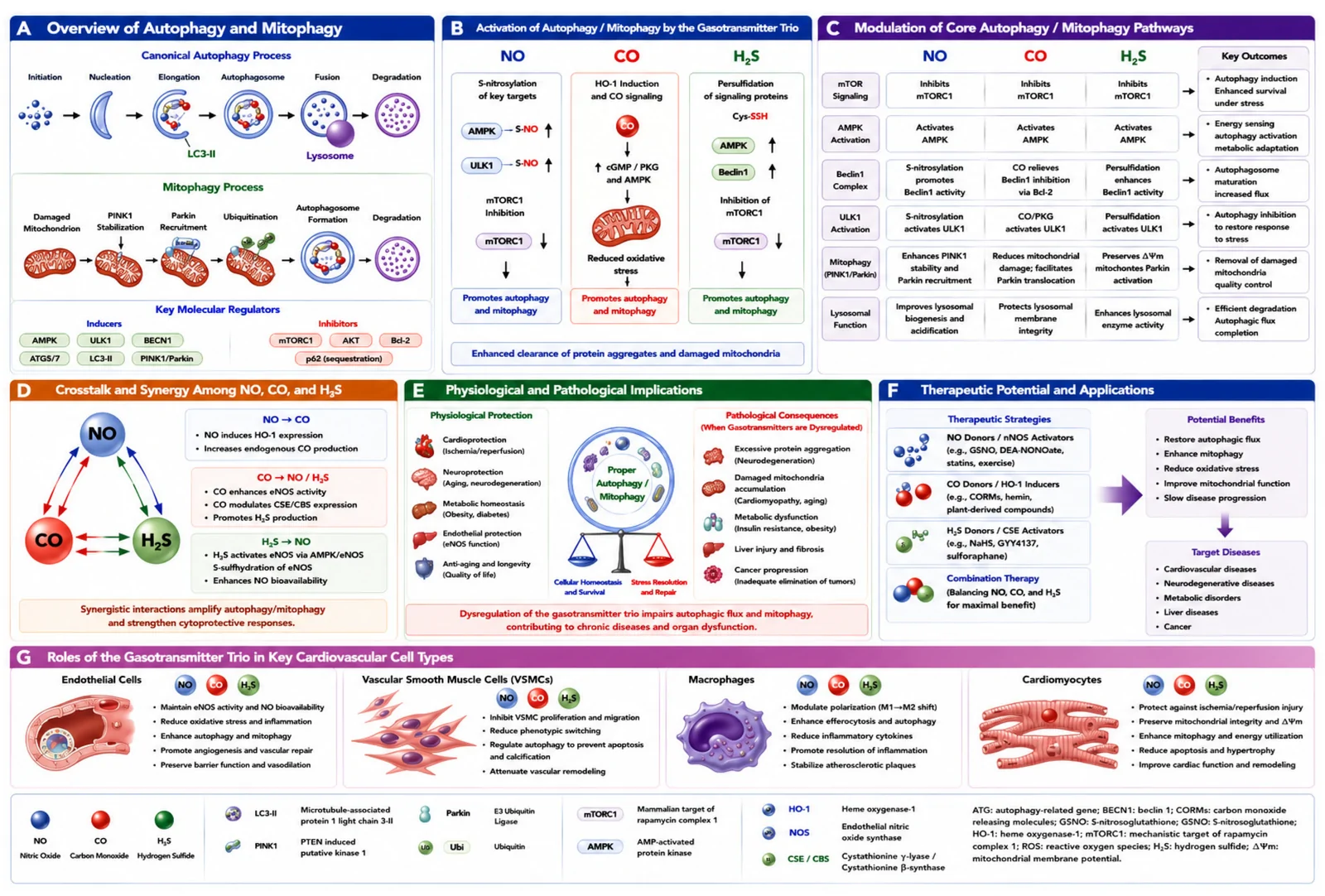
**Regulation of autophagy and mitophagy by the gasotransmitter trio (NO, CO, and H_2_S)**. This figure illustrates the mechanisms by which nitric oxide (NO), carbon monoxide (CO), and hydrogen sulfide (H_2_S) regulate autophagy and mitophagy and their implications in cardiovascular and metabolic diseases. (**A**) Overview of canonical autophagy and mitophagy. Autophagy involves initiation, nucleation, elongation, autophagosome formation, fusion with lysosomes, and cargo degradation. Mitophagy selectively clears damaged mitochondria via the PINK1/Parkin pathway. Key activators include AMPK, ULK1, Beclin-1, ATG proteins, and LC3-II, while mTORC1, AKT, and Bcl-2 act as major inhibitors. (**B**) Activation by individual gasotransmitters. NO promotes autophagy via S-nitrosylation of the AMPK/ULK1 pathways and suppression of mTORC1. CO, generated by HO-1, activates cGMP/PKG and AMPK signaling, reduces oxidative stress, and improves mitochondrial quality control. H_2_S induces protein persulfidation, activates AMPK/Beclin-1, inhibits mTORC1, and enhances autophagic flux and mitophagy. Together, they promote clearance of damaged proteins and dysfunctional mitochondria. (**C**) Modulation of core signaling networks. The gasotransmitter trio converges on key nodes (mTORC1, AMPK, ULK1, Beclin-1, PINK1/Parkin, and lysosomes) to enhance autophagosome formation, mitochondrial turnover, and cargo degradation. (**D**) Molecular crosstalk and synergy. Bidirectional interactions amplify responses: NO induces HO-1 and CO production; CO enhances eNOS and H_2_S biosynthesis; H_2_S activates eNOS/AMPK to increase NO bioavailability. This synergy strengthens cytoprotective autophagy under stress. (**E**) Physiological and pathological implications. Balanced gasotransmitter signaling supports cardioprotection, neuroprotection, metabolic homeostasis, endothelial function, and healthy aging by maintaining autophagic flux and mitochondrial quality control. Dysregulation impairs autophagy/mitophagy, leading to protein aggregation, mitochondrial dysfunction, fibrosis, and disease progression. (**F**) Therapeutic potential. Strategies include NO donors/eNOS activators, CO-releasing molecules (CORMs)/HO-1 inducers, H_2_S donors/CSE activators, and combination therapies. These enhance autophagy, mitophagy, and mitochondrial function, and may slow the progression of cardiovascular and metabolic disease. (**G**) Roles in cardiovascular cell types. In endothelial cells, the trio maintains vascular homeostasis, reduces oxidative stress, and promotes angiogenesis. In vascular smooth muscle cells, it inhibits proliferation, phenotypic switching, and calcification via autophagy. In macrophages, it drives M2 polarization, efferocytosis, and resolution of inflammation. In cardiomyocytes, it enhances mitophagy, preserves mitochondrial integrity, and protects against ischemia–reperfusion injury and heart failure.

Among the various forms of autophagy, mitophagy, the selective removal of damaged mitochondria, has emerged as a key determinant of cellular fate because mitochondria represent major sources of ROS and regulators of apoptosis, ferroptosis, and metabolic adaptation [93]. In contrast to ferroptosis, which culminates in irreversible cellular damage, autophagy and mitophagy function primarily as adaptive quality-control mechanisms that preserve cellular integrity under stress conditions. Emerging evidence indicates that NO, CO, and H_2_S regulate multiple stages of autophagic and mitophagic flux through coordinated modulation of nutrient-sensing pathways, mitochondrial signaling, and redox-sensitive post-translational modifications. The major molecular mechanisms through which the gasotransmitter trio orchestrates autophagy and mitophagy are summarized in Figure 5.

#### 4.2.1. Gasotransmitters Activate Cytoprotective Autophagy

A growing body of evidence indicates that all three gasotransmitters activate autophagic pathways through overlapping signaling mechanisms involving AMP-activated protein kinase (AMPK), mammalian target of rapamycin (mTOR), and transcription factor EB (TFEB) [46,94]. NO can induce autophagy by activating AMPK and modulating redox-sensitive signaling pathways [95]. Physiological NO signaling promotes the removal of damaged cellular components and improves resistance to ischemic and inflammatory stress. Similarly, low-dose CO stimulates autophagic flux through transient mitochondrial stress and activation of adaptive metabolic programs [96]. H_2_S has emerged as a particularly potent regulator of autophagy. Through persulfidation-dependent activation of AMPK and SIRT1 signaling, H_2_S promotes autophagic degradation and enhances cellular stress tolerance [97]. Furthermore, H_2_S-mediated activation of Nrf2 creates a coordinated antioxidant-autophagy response that facilitates recovery from oxidative injury [64].

#### 4.2.2. Regulation of Mitophagy and Mitochondrial Fitness

Increasing evidence suggests that the cytoprotective effects of gasotransmitters are strongly linked to their ability to preserve mitochondrial quality control [98]. NO regulates mitochondrial dynamics through reversible S-nitrosylation of proteins involved in mitochondrial fusion and fission, including Drp1 [59]. Under physiological conditions, these modifications facilitate the selective elimination of dysfunctional mitochondria and the maintenance of mitochondrial network integrity. H_2_S promotes mitophagy by activating the PINK1-Parkin pathway and enhancing mitochondrial biogenesis [99]. Simultaneously, H_2_S improves mitochondrial respiration efficiency and reduces ROS generation, thereby decreasing the burden of damaged mitochondria that require removal [90]. CO contributes to mitochondrial quality control by inducing mild mitochondrial stress, a process often described as mitohormesis [66,69]. Low concentrations of CO activate adaptive transcriptional programs that enhance mitochondrial biogenesis, antioxidant capacity, and stress resistance. These effects ultimately support mitochondrial turnover and improve cellular resilience.

#### 4.2.3. Gasotransmitter Control of the Ferroptosis-Mitophagy Balance

Recent studies increasingly suggest that ferroptosis and mitophagy are not independent processes but are interconnected components of mitochondrial quality control [100]. Excessive mitochondrial dysfunction may trigger ferroptosis through ROS accumulation and iron dysregulation, whereas efficient mitophagy removes damaged mitochondria and prevents progression toward irreversible cell death.

Within this framework, NO, CO, and H_2_S collectively function as upstream regulators of the ferroptosis-mitophagy axis. By suppressing lipid peroxidation, preserving mitochondrial integrity, and promoting adaptive autophagic responses, gasotransmitters shift cellular fate toward survival rather than death. This integrated mechanism appears particularly important in ischemia–reperfusion injury, neurodegenerative disorders, cardiovascular diseases, and metabolic syndromes, where oxidative stress and mitochondrial dysfunction represent central pathogenic events [46,47,101].

Taken together, the gasotransmitter trio acts as a sophisticated mitochondrial quality-control network that coordinates ferroptosis, autophagy, and mitophagy. Rather than serving solely as signaling molecules, NO, CO, and H_2_S function as master regulators of cellular adaptation, with the ultimate role of preserving mitochondrial fitness and determining cellular fate under physiological and pathological stress.

### 4.3. Gasotransmitter Dysregulation, Metabolic Disease, and Vascular Aging

The ferroptosis-, autophagy-, and mitophagy-regulating functions of NO, CO, and H_2_S described above are themselves subject to age- and metabolic disease- associated decline, establishing a mechanistic chain that links gasotransmitter biology directly to vascular aging and atherogenesis. Aging progressively reduces eNOS expression and activity in the vascular endothelium, driven by oxidative inactivation and reduced availability of substrates and cofactors [19,52]. In parallel, CSE expression and H_2_S-generating capacity decline with cellular senescence [64], while HO-1 induction becomes blunted in the chronic low-grade inflammatory state that characterizes obesity and metabolic syndrome, despite HO-1 ordinarily being the principal source of cytoprotective CO in vascular and adipose tissue [54]. The combined result is a coordinated decline in the bioavailability of NO, H_2_S, and CO, particularly in disease states such as diabetes, obesity, and metabolic syndrome, where cytoprotective gasotransmitter signaling is most needed.

This decline in bioavailability directly undermines the cell-fate mechanisms described in Section 4.1 and Section 4.2: diminished NO/H_2_S signaling impairs GPX4-dependent suppression of lipid peroxidation, lowering the threshold for ferroptosis, while reduced AMPK/PINK1-Parkin activation blunts autophagic and mitophagic clearance of damaged mitochondria [64,80]. The resulting accumulation of senescent, iron-loaded, and functionally impaired mitochondria within endothelial cells, vascular smooth muscle cells, and macrophages creates a self-reinforcing cycle: dysfunctional mitochondria generate further ROS and labile iron, which in turn suppress residual eNOS/CSE/HO-1 activity, accelerating vascular stiffening, endothelial senescence, and plaque progression. Consistent with this model, H_2_S supplementation restores cardioprotection specifically in diabetic and senescence-prone models [64,80], indicating that gasotransmitter decline is not merely a marker of metabolic and vascular aging but a modifiable contributor to it, and providing a mechanistic rationale for the gasotransmitter-based and multi-gas therapeutic strategies discussed in Section 5.

## 5. Next-Generation Smart Delivery Systems for Cardiovascular Gas Therapy

**Figure 6:**
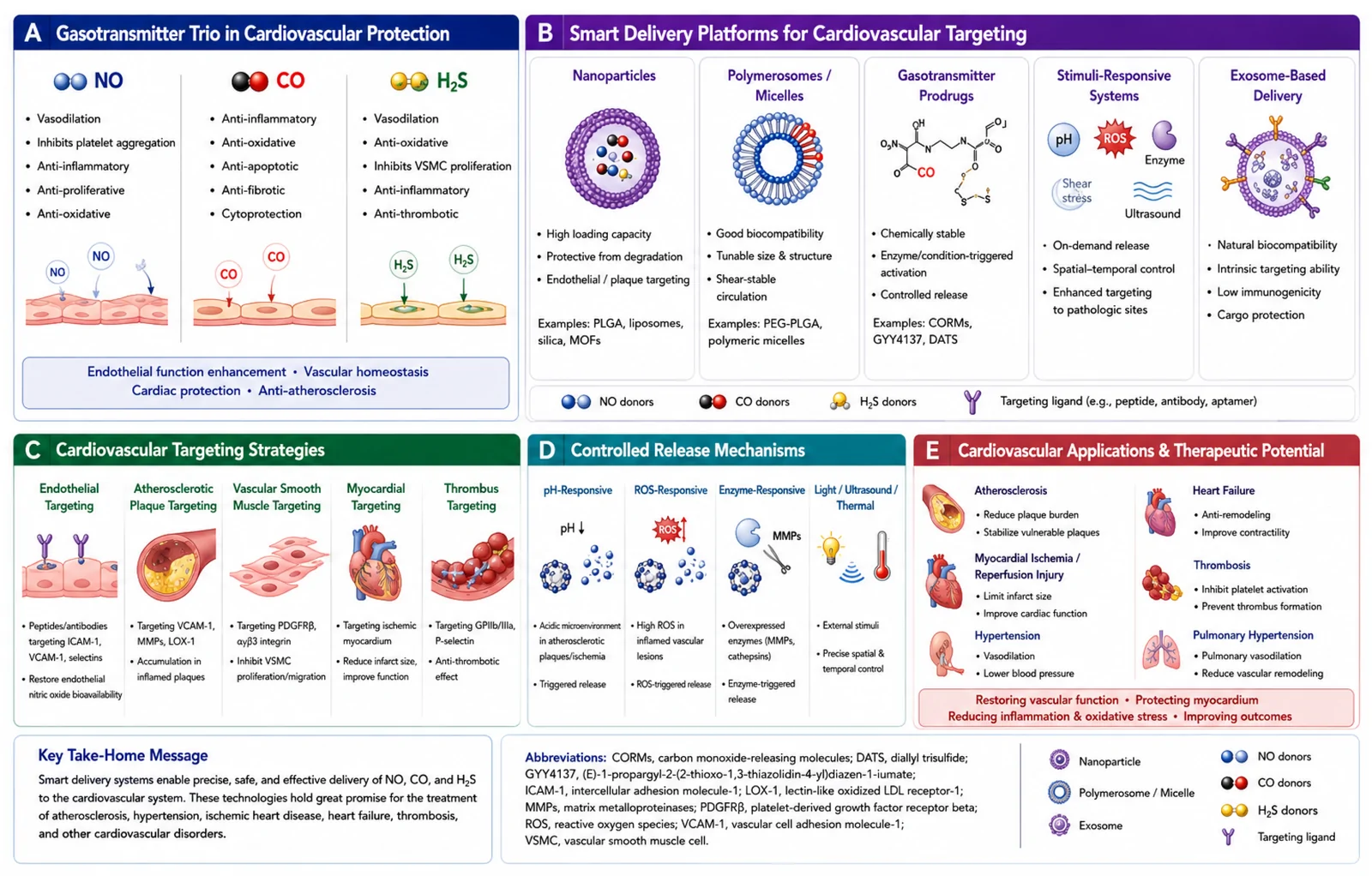
**Smart Delivery Systems for the Gasotransmitter Trio (NO, CO, H_2_S) in Cardiovascular Disease**. (**A**) Cardiovascular protective actions of individual gasotransmitters. NO primarily promotes vasodilation, inhibits platelet aggregation, suppresses vascular inflammation, and preserves endothelial function. CO exerts anti-inflammatory, antioxidant, anti-apoptotic, and cytoprotective effects by modulating mitochondrial function and stress signaling. H_2_S contributes to vasorelaxation, antioxidant defense, inhibition of vascular smooth muscle cell proliferation, anti-inflammatory responses, and antithrombotic activity. Collectively, these gasotransmitters maintain vascular homeostasis and protect against cardiovascular injury. (**B**) Representative smart delivery platforms for cardiovascular-targeted gasotransmitter administration, including nanoparticles, polymeric micelles/polymersomes, gasotransmitter prodrugs, stimuli-responsive systems, and exosome-based carriers. These platforms improve drug stability, prolong circulation time, enable controlled and site-specific release, and reduce systemic toxicity while enhancing delivery to diseased cardiovascular tissues. (**C**) Targeting strategies for precision cardiovascular therapy, including endothelial, atherosclerotic plaque, vascular smooth muscle cell, ischemic myocardium, and thrombus targeting via disease-specific ligands or pathological biomarkers. Such approaches increase therapeutic accumulation at lesion sites while minimizing off-target effects. (**D**) Controlled-release mechanisms that exploit pathological microenvironments characteristic of cardiovascular disease, including acidic pH, elevated reactive oxygen species (ROS), disease-associated enzymes (e.g., matrix metalloproteinases and cathepsins), and externally applied stimuli such as light, ultrasound, or thermal activation, thereby enabling spatiotemporally regulated gasotransmitter release. (**E**) Major cardiovascular applications of gasotransmitter-based nanomedicine, including atherosclerosis, myocardial ischemia–reperfusion injury, hypertension, heart failure, thrombosis, and pulmonary hypertension. Through restoration of endothelial function, suppression of oxidative stress and inflammation, modulation of vascular remodeling, inhibition of platelet activation, and preservation of mitochondrial homeostasis, smart delivery systems substantially enhance the therapeutic potential of NO-, CO-, and H_2_S-based interventions while reducing systemic adverse effects. Collectively, these next-generation delivery technologies provide a promising translational strategy for precision cardiovascular medicine by integrating targeted delivery, controlled release, and disease-responsive activation to maximize therapeutic efficacy and safety. Collectively, these advanced delivery platforms are transforming gasotransmitter research from fundamental signaling biology into a clinically translatable strategy for precision cardiovascular medicine. Future smart nanomedicines capable of disease-responsive, spatiotemporally controlled, and multi-gas release may enable personalized modulation of the gasotransmitter network to treat complex cardiovascular disorders, thereby maximizing therapeutic efficacy while minimizing systemic toxicity. CORM, carbon monoxide-releasing molecule; EPR, enhanced permeability and retention; GSH, glutathione; MOF, metal–organic framework; NORM, nitric oxide-releasing molecule; PLGA, poly(lactic-co-glycolic acid); ROS, reactive oxygen species; TPP^+^, triphenylphosphonium.

Recent advances in chemical biology and nanomedicine have accelerated the development of next-generation gasotransmitter delivery platforms. Compared with conventional gas donors, these systems provide improved stability, enhanced tissue targeting, and controlled spatiotemporal gas release, thereby overcoming major limitations associated with the short half-lives and dose-dependent toxicity of NO, CO, and H_2_S [102,103,104]. These innovations have transformed gasotransmitter research from simple donor-based pharmacology to precision gas medicine, in which programmable delivery systems can recapitulate endogenous gas signaling dynamics. The therapeutic potential of NO, CO, and H_2_S is constrained by physicochemical properties that differ fundamentally from conventional drugs: extremely short biological half-lives, narrow therapeutic windows, and dose-limiting systemic toxicity [10]. In the cardiovascular context specifically, these limitations are consequential because the trio-network effects described in Section 3 and Section 4—reciprocal enzymatic regulation, ferroptosis suppression, and mitophagy activation—depend on sustained, localized gasotransmitter concentrations that free-gas administration cannot achieve. Smart delivery systems have therefore emerged as a prerequisite for translating the molecular crosstalk mechanisms discussed above into cardiovascular therapeutics, rather than as a stand-alone engineering topic. Representative donor and nanoplatform categories, together with their major advantages, limitations, and clinical bottlenecks, are summarized in Figure 6.

### 5.1. Why Free Gasotransmitter Administration Fails in Cardiovascular Disease

Three factors limit direct administration of NO, CO, or H_2_S in cardiovascular therapy. First, half-lives are measured in seconds to minutes: NO is scavenged by hemoglobin and reactive thiols, H_2_S is rapidly oxidized in the mitochondria, and CO is redistributed via heme-containing proteins, so systemic levels fall before reaching diseased myocardium or vasculature [10,19,24,31]. Second, the effective dose is close to the toxic dose—excess NO causes profound hypotension and nitrosative stress, excess CO impairs oxygen transport via carboxyhemoglobin formation, and excess H_2_S inhibits cytochrome c oxidase, which is precisely the bidirectional concentration-dependence described in Section 3.2 [1,10,28,29]. Third, free gases lack tissue specificity, so a dose sufficient to modulate diseased vasculature or myocardium inevitably exposes healthy organs [102]. These constraints, rather than any single technical limitation, are what motivate the platform categories in Table 5.

### 5.2. Stimuli-Responsive Donors and Nanoplatforms: Cardiovascular Applications

Donor and nanocarrier chemistries (NORMs, CORMs, H_2_S donors, MOFs, polymeric micelles, liposomes, and biomimetic carriers) share a common design logic protecting a reactive gas during circulation and releasing it in response to a disease-associated trigger (pH, ROS, glutathione, enzymatic activity, or an external stimulus such as light) and their individual mechanisms, advantages, and limitations are detailed in Table 5 rather than repeated here. In cardiovascular applications specifically, this design logic has enabled: photoresponsive and enzyme-triggered NO/CO donors for spatially restricted vasodilation and anti-inflammatory signaling in atherosclerotic plaque without systemic hypotension [105,106,109,110]; slow-releasing and ROS-responsive H_2_S donors (e.g., GYY4137, AP39) that recapitulate sustained physiological H_2_S tone for cardioprotection in ischemia–reperfusion models, in contrast to instantaneous-release sulfide salts [111,112,113,114]; and MOF- and liposome-based nanocarriers that exploit the acidic, ROS-rich microenvironment of ischemic or atherosclerotic tissue for targeted, sustained release [115,116,117,118,119,120]. These CV-specific outcomes, rather than the underlying materials chemistry, are the translational rationale for the platform categories in Table 5.

### 5.3. Multi-Gas Nanocarriers for Combinatorial Regulation of Cardiovascular Cell Fate

We define multi-gas nanocarriers as delivery platforms that co-encapsulate two or more gasotransmitter donors or one donor together with a redox-active or iron-chelating payload within a single carrier for spatiotemporally coordinated release, in contrast to the single-gas donor systems in Table 5. Because NO, CO, and H_2_S function as an integrated network rather than as independent mediators (Section 3 and Section 4), such platforms may more accurately recapitulate physiological signaling and directly target all three cell-fate programs discussed in Section 4. Combined NO/H_2_S release generates hybrid signaling species (SSNO^−^, HNO) that amplify cytoprotective autophagic flux and vasodilatory responses beyond either gas alone, and more effectively restores GPX4 activity to limit ferroptotic lipid peroxidation, consistent with the gasotransmitter-mediated redox and mitochondrial quality-control mechanisms described in Section 4 [36,69,71,90]; combined CO-mediated mitohormesis and H_2_S-driven antioxidant signaling likewise act synergistically, rather than additively, on mitophagy and mitochondrial quality control [69,90]. Dual and multi-responsive MOFs, core–shell/Janus nanoparticles, co-loaded liposomes/micelles, and hybrid CORM–NORM–H_2_S-donor conjugates now provide the technical means to co-encapsulate and independently trigger release of these combinations within a single carrier [115,116,117,122]. Clinically, the scenarios in which combinatorial release is most directly justified are those where Section 4 identifies concurrent ferroptosis, autophagy, and mitophagy dysregulation as central to pathology such as ischemia–reperfusion injury, atherosclerotic plaque instability, and diabetic vasculopathy, where simultaneous restoration of NO-, CO-, and H_2_S-dependent cytoprotection is unlikely to be achievable by any single-gas platform. Multi-gas co-delivery therefore represents the logical convergence of the delivery technologies in Table 5 with the crosstalk and cell-fate biology established in Section 3 and Section 4, and is the direction we anticipate will dominate the next generation of gasotransmitter-based cardiovascular therapeutics.

## 6. Conclusions and Future Perspectives

**Figure 7:**
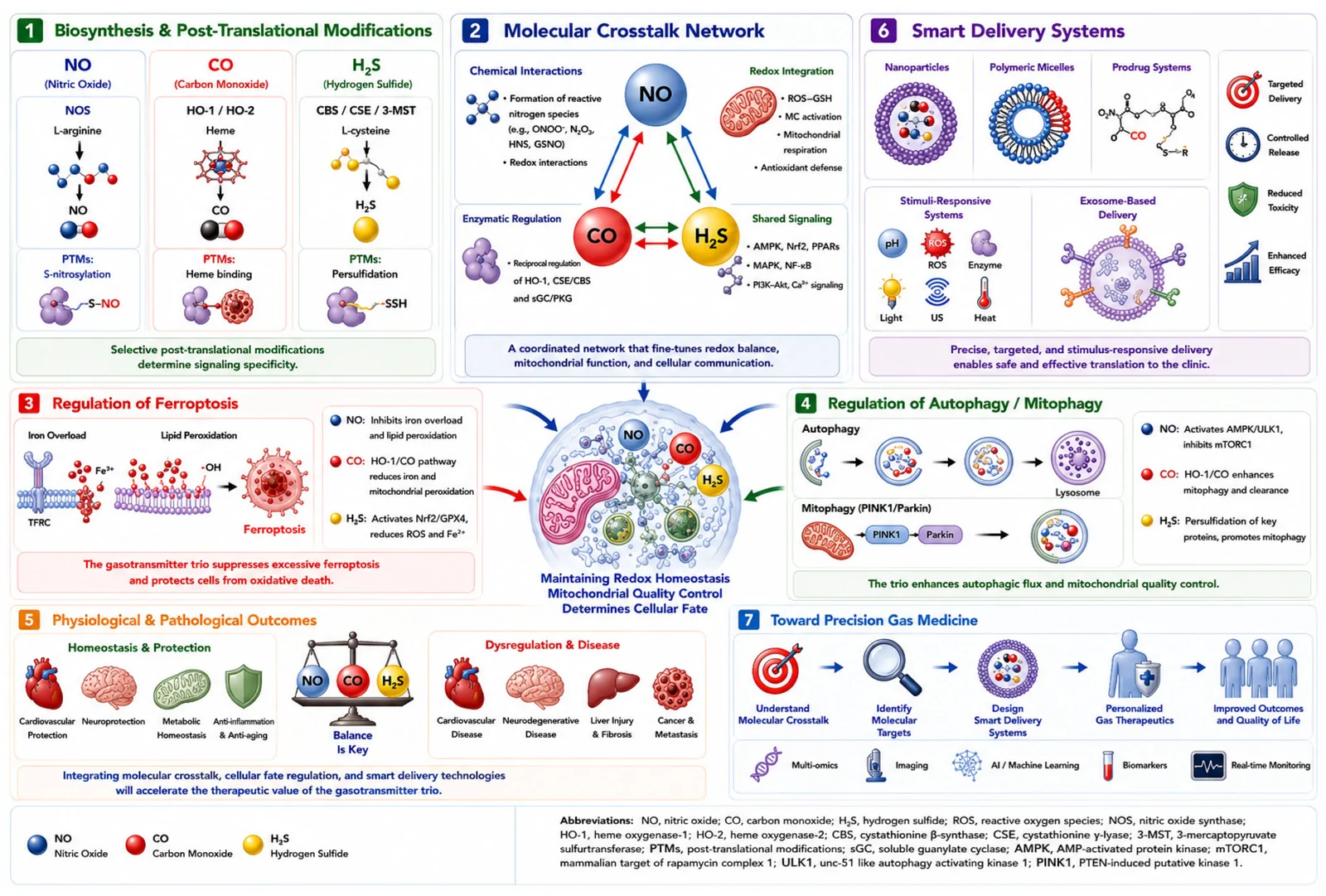
**Toward precision cardiovascular gas medicine: integrating molecular crosstalk, cellular fate regulation, and smart delivery technologies for the gasotransmitter trio (NO, CO, and H_2_S).** This figure illustrates a future framework for precision gas medicine in which nitric oxide (NO), carbon monoxide (CO), and hydrogen sulfide (H_2_S) are viewed as components of an integrated signaling network rather than individual therapeutic agents. (**1**) Biosynthesis and post-translational modifications (PTMs). Endogenous production of NO, CO, and H_2_S is mediated by nitric oxide synthases (NOS), heme oxygenases (HO-1/HO-2), and sulfur-metabolizing enzymes including cystathionine β-synthase (CBS), cystathionine γ-lyase (CSE), and 3-mercaptopyruvate sulfurtransferase (3-MST), respectively. These gasotransmitters exert their biological actions through S-nitrosylation, heme-dependent coordination, and persulfidation, thereby modulating protein function and signaling specificity. (**2**) Molecular crosstalk network. NO, CO, and H_2_S engage in direct chemical interactions, reciprocal enzymatic regulation, and shared redox signaling pathways. These interactions generate hybrid signaling intermediates and coordinate key pathways involving sGC–cGMP signaling, Nrf2 activation, mitochondrial adaptation, inflammatory regulation, and cellular stress responses. (**3**) Regulation of ferroptosis. The gasotransmitter trio collectively suppresses excessive ferroptosis by modulating iron metabolism, inhibiting lipid peroxidation, activating antioxidant defense systems, and preserving GPX4 activity. Balanced gasotransmitter signaling protects tissues from oxidative injury and ferroptotic cell death. (**4**) Regulation of autophagy and mitophagy. NO, CO, and H_2_S promote mitochondrial quality control by activating AMPK–ULK1 signaling, inhibiting mTORC1, enhancing PINK1–Parkin-dependent mitophagy, and maintaining lysosomal function. These adaptive mechanisms facilitate removal of damaged mitochondria and support cellular survival. (**5**) Physiological and pathological outcomes. Homeostatic gasotransmitter signaling contributes to cardiovascular protection, neuroprotection, metabolic balance, anti-inflammatory responses, and healthy aging. In contrast, disruption of the gasotransmitter network promotes oxidative stress, mitochondrial dysfunction, inflammation, fibrosis, neurodegeneration, metabolic disorders, and cancer progression. (**6**) Smart delivery systems. Emerging nanotechnologies, including nanoparticles, liposomes, polymeric micelles, metal–organic frameworks (MOFs), stimuli-responsive carriers, and exosome-based systems, enable controlled, targeted, and programmable delivery of gasotransmitters. These platforms improve therapeutic efficacy while minimizing systemic toxicity. (**7**) Future precision gas medicine. Integration of gasotransmitter biology with redox proteomics, spatial multi-omics, artificial intelligence-assisted biomarker discovery, and programmable multi-gas delivery systems may enable personalized gasotherapeutics. Future therapeutic strategies are expected to combine real-time disease sensing with spatiotemporally controlled release of NO, CO, and H_2_S, thereby optimizing treatment outcomes in cardiovascular, neurodegenerative, inflammatory, metabolic, and malignant diseases. The central concept highlighted in this figure is that cellular fate is determined by coordinated regulation of redox homeostasis and mitochondrial quality control. Future precision gas medicine will therefore depend on the ability to quantitatively monitor and therapeutically modulate the gasotransmitter trio network in a disease-specific and patient-specific manner. AI, artificial intelligence; AMPK, AMP-activated protein kinase; CBS, cystathionine β-synthase; CSE, cystathionine γ-lyase; GPX4, glutathione peroxidase 4; HO, heme oxygenase; MOF, metal–organic framework; mTORC1, mechanistic target of rapamycin complex 1; Nrf2, nuclear factor erythroid 2-related factor 2; NOS, nitric oxide synthase; PINK1, PTEN-induced kinase 1; PTM, post-translational modification; sGC, soluble guanylate cyclase; ULK1, unc-51-like kinase 1; 3-MST, 3-mercaptopyruvate sulfurtransferase.

As discussed throughout this review, the biological functions of NO, CO, and H_2_S extend far beyond their traditional roles as individual gaseous mediators. Emerging evidence indicates that these gasotransmitters operate as an integrated signaling network that coordinates redox homeostasis, mitochondrial quality control, cellular fate determination, and tissue adaptation. At the same time, rapid advances in biomaterials engineering, smart delivery technologies, and precision medicine are creating new opportunities to translate gasotransmitter biology into clinically actionable therapeutic strategies. To integrate these concepts into a unified framework, Figure 7 summarizes the emerging paradigm of precision gas medicine, highlighting the interconnected relationships among gasotransmitter crosstalk, ferroptosis, autophagy, smart delivery systems, and future personalized therapeutics.

### 6.1. From Individual Gasotransmitters to an Integrated Trio Network

Over the past four decades, NO, CO, and H_2_S have undergone a remarkable conceptual transformation from toxic environmental gases to indispensable endogenous signaling molecules [3,8,12,14]. Extensive research has established their fundamental roles in regulating vascular homeostasis, inflammation, metabolism, mitochondrial function, and tissue repair. Nevertheless, much of the existing literature has traditionally examined these gasotransmitters as independent signaling entities.

The evidence summarized in this review supports a different perspective. Rather than functioning in isolation, NO, CO, and H_2_S form a highly interconnected signaling network characterized by direct chemical interactions, reciprocal enzymatic regulation, and convergence on common downstream pathways [35,36,37,38,82]. Through mechanisms such as S-nitrosylation, persulfidation, and heme-dependent signaling, the gasotransmitter trio continuously exchanges biochemical information and collectively orchestrates cellular adaptation to environmental and metabolic stress. This integrated framework may be conceptualized as a “Gasotransmitter Trio Network,” in which biological outcomes are determined not by the activity of a single gasotransmitter but by the dynamic balance among all three signaling systems. Such a network perspective provides a more comprehensive explanation for the diverse and sometimes paradoxical biological effects observed in cardiovascular disease, neurodegeneration, cancer, inflammation, and metabolic disorders.

### 6.2. Gasotransmitters as Master Regulators of Cellular Fate

One of the most important advances in contemporary gasotransmitter biology is the recognition that these molecules influence fundamental cell fate decisions. Emerging evidence demonstrates that NO, CO, and H_2_S regulate ferroptosis, autophagy, and mitophagy through coordinated modulation of redox signaling, iron metabolism, mitochondrial dynamics, and antioxidant defense systems [33,39,40,41,43,92].

In particular, the gasotransmitter trio appears to function as a mitochondrial quality-control network. NO-mediated S-nitrosylation, H_2_S-mediated persulfidation, and CO-induced mitohormetic signaling converge upon pathways that preserve mitochondrial integrity and maintain bioenergetic homeostasis [59,64,66,90]. By balancing ferroptotic susceptibility and mitophagic clearance, these gases collectively determine whether cells adapt, survive, or undergo regulated death under pathological conditions.

This emerging paradigm shifts the focus of gasotransmitter research beyond classical vasodilation and neurotransmission toward a broader role in cellular quality control. Such a perspective may help explain why gasotransmitter dysregulation contributes to diverse diseases that share common pathological features, including oxidative stress, mitochondrial dysfunction, chronic inflammation, and metabolic reprogramming.

### 6.3. Current Challenges and Unresolved Questions

Despite substantial progress, several major challenges continue to impede the clinical translation of gasotransmitter biology.

First, accurate quantification of endogenous gasotransmitter concentrations remains technically difficult. Because NO, CO, and H_2_S are highly reactive, diffusible, and rapidly metabolized, precise measurement of their tissue-specific concentrations remains challenging [123]. Current analytical approaches often lack sufficient spatial and temporal resolution to capture dynamic signaling events occurring within subcellular compartments. The proposed direction: real-time, tissue-resolved precision gas-monitoring systems that combine fluorescent probes, electrochemical biosensors, and wearable sensing devices (Table 6).

Second, the therapeutic window of gasotransmitters remains incompletely defined. Physiological concentrations generally confer cytoprotective effects, whereas excessive accumulation may induce toxicity [10,28,29]. The transition point between beneficial and harmful signaling likely varies across tissues, disease states, and patient populations. The proposed direction: adaptive precision gas medicine built on programmable release systems and AI-assisted, pharmacokinetically informed dose prediction (Table 6).

Third, the complexity of gasotransmitter crosstalk remains incompletely understood. Although substantial progress has been made in characterizing NO-H_2_S interactions, the broader signaling network involving CO, reactive sulfur species, reactive nitrogen species, and mitochondrial metabolites remains poorly defined [33,36,70]. Systems biology approaches integrating redox proteomics, metabolomics, spatial transcriptomics, and computational modeling will likely be required to decipher these multilayered interactions. The proposed direction: AI-assisted multi-omics integration and digital twin modeling to resolve network-level crosstalk that single-pathway studies cannot capture (Table 6).

Finally, translation from experimental models to human disease remains challenging. Many mechanistic insights have been derived from in vitro systems or animal models, whereas clinical evidence remains comparatively limited. Bridging this translational gap will require carefully designed clinical studies supported by advanced biomarker platforms and precision-delivery technologies. The proposed direction: GMP-compliant, scalable gasotransmitter manufacturing and regulatory harmonization to enable evidence-based clinical translation (Table 6).

Each of these four challenges, together with its corresponding emerging strategy and future direction, is systematically cross-referenced in Table 6.

### 6.4. Future Outlook: Toward Precision Gas Medicine 2.0

Looking forward, the future of gasotransmitter therapeutics will likely be shaped by the convergence of molecular medicine, nanotechnology, artificial intelligence, and biomaterials engineering [115,121]. Collectively, the advances discussed throughout this review support a transition from traditional single-gas pharmacology toward a systems-level framework of precision gas medicine. In this emerging paradigm, endogenous gasotransmitter biology, cellular fate regulation, and smart delivery technologies are integrated to achieve personalized therapeutic modulation of disease-associated signaling networks. Future implementation of this framework will require the convergence of biomarker-guided diagnosis, precision delivery systems, and quantitative monitoring of gasotransmitter dynamics across diverse pathological conditions. Despite remarkable progress, several critical challenges remain before gasotransmitter-based therapies can achieve widespread clinical implementation. These include accurate quantification of endogenous gasotransmitter levels, optimization of therapeutic dosing, development of tissue-specific delivery systems, identification of predictive biomarkers, and validation of long-term safety profiles. The major translational challenges and future opportunities are summarized in Table 6.

As summarized in Table 6, the future of gasotransmitter research is expected to move beyond the administration of individual gaseous mediators toward integrated therapeutic platforms that reproduce endogenous gasotransmitter networks. Advances in nanotechnology, biomaterials engineering, molecular diagnostics, and multi-omics approaches will likely facilitate the development of personalized gasotransmitter-based interventions for diverse human diseases.

Importantly, the convergence of gasotransmitter biology with precision medicine may enable a paradigm shift from reactive disease management toward proactive modulation of cellular fate. By integrating biomarker-guided diagnosis, targeted delivery systems, and dynamic monitoring of redox homeostasis, future gasotransmitter therapeutics may achieve unprecedented levels of efficacy and safety.

A critical prerequisite for realizing this vision is the development of technologies capable of delivering gasotransmitters with high spatial and temporal precision. The development of stimuli-responsive donors and programmable nanocarriers has already demonstrated the feasibility of achieving spatiotemporally controlled gas release [102,103,104,105,106,107,108,109,110,111,112,113,114,115,116,117,118,119,120,121,122]. Future delivery systems may incorporate real-time biosensing capabilities, enabling autonomous regulation of gasotransmitter release in response to local pathological signals such as oxidative stress, hypoxia, acidosis, or inflammation. Equally exciting is the emergence of multi-gas therapeutic platforms. Because endogenous gasotransmitters function as an integrated signaling network, in which NO, CO, and H_2_S exhibit extensive biochemical crosstalk and reciprocal regulation, simultaneous and coordinated modulation of these gaseous mediators may more accurately recapitulate physiological signaling than the administration of individual gases [35,73,80]. Recent advances in nanotechnology, biomaterials engineering, and stimuli-responsive delivery systems have further enabled the development of programmable platforms capable of controlled, localized, and potentially multi-gas release [115,116,117,118,119,120,121,122]. Such next-generation gas delivery systems may enable dynamic modulation of ferroptosis, autophagy, mitochondrial quality control, and immune responses according to disease-specific requirements.

Beyond advances in delivery technologies, emerging redox proteomics, spatial multi-omics, and single-cell profiling approaches may further facilitate personalized gas medicine by identifying patient-specific gasotransmitter signatures, signaling vulnerabilities, and therapeutic response patterns [124]. In this scenario, gasotransmitter therapies would no longer rely on empirical dosing but instead become guided by molecular profiling and precision biomarker monitoring. Ultimately, the integration of systems biology, advanced biomaterials, and precision diagnostics may transform gasotransmitter therapeutics from broadly acting interventions into highly personalized strategies tailored to individual disease contexts.

In conclusion, NO, CO, and H_2_S should no longer be viewed merely as independent gaseous mediators. Rather, they constitute a dynamic and highly coordinated signaling network that governs cellular adaptation, mitochondrial fitness, and cell fate decisions. Continued integration of gasotransmitter biology with advanced delivery technologies will likely usher in a new era of precision gas medicine, offering transformative therapeutic opportunities for cardiovascular, neurodegenerative, inflammatory, metabolic, and malignant diseases.

### 6.5. Core Paradigm and Priorities for the Next Three to Five Years

The central paradigm advanced throughout this review can be condensed as follows: NO, CO, and H_2_S do not act as three independent gaseous mediators. Rather, they constitute a concentration-sensitive “gasotransmitter trio network” that reciprocally regulates its own biosynthetic enzymes (CBS, CSE, HO, and NOS) and converges on shared effectors, including Nrf2, sGC–cGMP signaling, and mitochondrial quality-control pathways. Through this integrated network, the gasotransmitter trio coordinately governs the ferroptosis–autophagy–mitophagy axis that determines cardiovascular cell fate. Physiological concentrations of this network sustain vascular homeostasis, whereas its pathological dysregulation, accentuated by aging and metabolic disease, drives ferroptotic and mitochondrial injury underlying atherosclerosis, ischemia–reperfusion injury, and heart failure. Realizing the therapeutic potential of this paradigm now depends less on further mechanistic discovery with single gases and more on the technological and clinical priorities outlined below.

Based on the gaps identified throughout this review, we propose the following five research priorities for the field over the next three to five years: (1) Real-time, tissue-resolved multi-gas biosensors capable of simultaneously quantifying NO, CO, and H_2_S in vivo, to finally resolve the spatial and temporal dynamics that current analytical methods cannot capture (Section 6.3). (2) Mechanistic definition of the concentration thresholds separating physiological (activating) from pathological (inhibitory) gasotransmitter signaling at each of the CBS/CSE/HO/NOS nodes (Section 3.2), to enable rational dosing rather than empirical titration. (3) First-in-human validation of multi-gas co-delivery nanoplatforms (Section 5.3), moving beyond preclinical proof-of-concept toward GMP-compliant manufacturing and early-phase clinical trials in ischemia–reperfusion injury or atherosclerotic disease. (4) Biomarker-guided patient stratification for gasotransmitter-based therapy, leveraging redox proteomics and metabolomics to identify which patients, and which disease stages, are most likely to benefit from gasotransmitter-targeted intervention. (5) Systems-biology and AI integration of multi-omics gasotransmitter signatures, to model the trio network computationally and predict individualized therapeutic windows rather than relying on single-pathway extrapolation. Collectively, progress on these five priorities would transform the gasotransmitter trio network from a well-characterized mechanistic framework into a clinically actionable therapeutic paradigm.

## Figures and Tables

**Table 1:** **Comparative Overview of Biosynthesis, Molecular Targets, and Signaling Mechanisms of NO, CO, and** **H_2_S.**

Feature	NO	CO	H_2_S
Major biosynthetic enzymes	eNOS, nNOS, iNOS	HO-1, HO-2	CBS, CSE, 3-MST
Principal substrate	L-arginine	Heme	L-cysteine and sulfur-containing amino acids
Major signaling mechanism	S-nitrosylation; sGC–cGMP activation	Heme coordination; modulation of heme proteins	Persulfidation/sulfhydration
Representative molecular targets	sGC, Drp1, NF-κB, mitochondrial proteins	sGC, cytochrome c oxidase, cytochrome P450 enzymes, globins	Keap1, KATP channels, mitochondrial enzymes, antioxidant proteins
Major biological effects	Vasodilation, antiplatelet activity, neurotransmission, and immune regulation	Anti-inflammation, mitochondrial adaptation, cytoprotection	Antioxidant defense, vasorelaxation, mitochondrial bioenergetics, stress adaptation
Pathological risk when excessive	Nitrosative stress, peroxynitrite formation, mitochondrial dysfunction	Hypoxia, mitochondrial inhibition, potential iron dysregulation	Inhibition of cytochrome c oxidase, mitochondrial toxicity
Key references	[19,20,21,31,58,59,60,61]	[8,9,10,18,53,54]	[25,33,55,56,62,63,64,65]

**Table 2:** **Molecular crosstalk mechanisms among NO, CO, and** **H_2_S.**

Interaction	Major Mechanism	Representative Outcome	Key References
NO–H_2_S	Formation of sulfur–nitrogen hybrid species, including SSNO^−^	Sustained NO-like signaling and prolonged vasorelaxation	[36,37,70]
NO–H_2_S	Generation of nitroxyl (HNO)	Cardioprotection and positive inotropic effects	[71,72]
H_2_S → NO	Enhancement of eNOS phosphorylation, dimerization, and NO bioavailability	Improved endothelial function and vasorelaxation	[35,38,73,74,75,76]
CO → NO	Modulation of endothelial NO release and eNOS-related signaling	Vasodilation and vascular adaptation	[77]
NO → CO	Induction of HO-1 expression through redox- and stress-responsive transcriptional pathways	Increased endogenous CO generation and cytoprotection	[78,79]
H_2_S → CO	Nrf2-dependent HO-1 induction	Antioxidant defense and stress adaptation	[64,80]
NO/CO/H_2_S convergence	Shared regulation of sGC–cGMP–PKG signaling	Vascular relaxation, antiplatelet activity, cardioprotection	[19,67,81]
NO/CO/H_2_S convergence	Regulation of KATP channels, Nrf2, NF-κB, HIF-1α, and mitochondrial pathways	Cytoprotection, redox balance, inflammation control	[22,64,68,80,82,83,84]

**Table 3:** **Bidirectional Regulation Among CBS, CSE, HO, and** **NOS.**

Enzyme Pair	Physiological (Activating) Effect	Pathological/High-Concentration (Inhibitory) Effect	Proposed Mechanism	Key References
H_2_S → NOS	Enhances eNOS phosphorylation (Ser1177), dimerization, BH_4_ preservation	Suppresses NOS activity	Cofactor interaction/redox alteration	[73,74,75,76]
NO → NOS (self)	—	Product inhibition of eNOS	S-nitrosylation-induced dimer collapse (loss of zinc-tetrathiolate cluster)	[52]
CO → CBS	— (CO not a physiological CBS activator)	Blocks CBS catalytic activity	Heme binding displaces Cys ligand; competitive with homocysteine	[18]
NO → CSE	—	Suppresses H_2_S-generating activity	S-nitrosation of Cys229	[26]
CO → eNOS	Activates eNOS, enhances NO production	—	PI3K/Akt-dependent signaling	[77]
NO → HO-1	Induces HO-1 transcription (Nrf2/HIF-1α), concentration-dependent increase	—	ARE/Nrf2-dependent transcription	[54,78,79]
H_2_S → HO-1	Induces HO-1 via Nrf2	—	Persulfidation of Keap1	[80]
NO → heme-protein maturation (general)	Facilitates heme incorporation into apo-hemoproteins at low levels	Reversibly inhibits heme insertion into apo-hemoproteins	Direct NO-heme binding competes with apoprotein maturation	[53]

**Table 4:** **Regulatory roles of NO, CO, and H_2_S in ferroptosis, autophagy, and** **mitophagy.**

Gasotransmitter	Effects on Ferroptosis	Effects on Autophagy/Mitophagy	Major Pathways or Targets	Disease Relevance	Key References
NO	Suppresses lipid peroxidation by terminating lipid radical chain reactions; preserves GPX4 activity under physiological conditions	Regulates autophagy through redox signaling and S-nitrosylation-dependent mechanisms	Lipid peroxyl radicals, GPX4, ferritin, Drp1, AMPK-related pathways	Cardiovascular injury, neurodegeneration, oxidative stress	[31,58,86,87]
CO	Context-dependent; moderate HO-1/CO signaling is protective, whereas excessive HO-1 activity may increase labile iron and promote ferroptosis	Activates autophagy through mitochondrial ROS and adaptive stress signaling	HO-1, biliverdin/bilirubin, Fe^2+^, cytochrome c oxidase, mitochondrial ROS	Ischemia–reperfusion injury, cancer, inflammation	[9,22,88,89]
H_2_S	Strongly inhibits ferroptosis by preserving GSH homeostasis, activating Nrf2, and maintaining GPX4 activity	Promotes autophagy and mitophagy through persulfidation, AMPK activation, and mitochondrial protection	Keap1–Nrf2, System Xc^−^, GSH, GPX4, mitochondrial enzymes	Cardiomyocyte injury, sepsis-associated injury, metabolic disease	[33,43,64,90,91,92]
Integrated trio network	Coordinately limits iron-driven lipid peroxidation and maintains redox balance	Supports mitochondrial quality control and cellular adaptation	Nrf2, GPX4, AMPK–mTOR, PINK1–Parkin, mitochondrial homeostasis	Cardiovascular, neurodegenerative, hepatic, renal, inflammatory, and malignant diseases	[39,40,41,42,43,44,45,93,94,95,96,97,98]

**Table 5:** **Representative gasotransmitter donors and smart delivery platforms for therapeutic** **applications.**

Platform/Donor Type	Gas Delivered	Trigger or Release Mechanism	Major Advantages	Major Limitations	Clinical/Translational Bottleneck	Representative Applications	Key References
Organic NO donors (NONOates, etc.)	NO	Spontaneous, pH-dependent, or chemical decomposition	Rapid NO release; useful experimental tools	Burst release kinetics; short half-life; tolerance with repeated dosing	Poor spatial control leads to systemic hypotension; limited disease-site selectivity	Vascular regulation, wound healing, antimicrobial therapy	[102,103,104]
Light-responsive NO-releasing materials	NO	Photoactivation	Spatial and temporal control of NO release	Requires external light source; limited tissue penetration of activating wavelengths	Impractical for deep/internal cardiovascular targets without invasive light delivery	Local vascular modulation, cancer therapy, biomaterials	[105,106]
CORMs	CO	Chemical or ligand-exchange release	Controlled CO administration without inhaled CO exposure	Off-target reactivity of metal scaffold; some CO release independent of intended trigger	Metal byproduct toxicity/clearance concerns; inconsistent release kinetics across compartments	Anti-inflammatory therapy, cytoprotection, cancer therapy	[10,107,108]
PhotoCORMs	CO	Light-triggered CO release	On-demand and localized CO delivery	Same light-penetration constraint as above; photobleaching limits repeat dosing	Not yet validated for non-superficial cardiovascular lesions	Cancer therapy, inflammatory diseases, mechanistic studies	[109]
Enzyme-triggered CORMs	CO	Disease- or enzyme-responsive activation	Improved selectivity and reduced systemic exposure	Dependent on disease-specific enzyme expression, variable between patients	Patient-to-patient variability in trigger-enzyme activity complicates dose standardization	Targeted CO therapy	[110]
Slow-releasing H_2_S donors (GYY4137-type)	H_2_S	Hydrolysis or slow chemical release	Sustained H_2_S exposure and reduced toxicity	Slow onset limits utility in acute settings; hydrolysis rate hard to fine-tune in vivo	Long-term safety and optimal dosing regimens undefined in humans	Cardioprotection, anti-inflammatory therapy, metabolic disease	[111,112,113,114]
pH- and ROS-responsive H_2_S donors	H_2_S	Acidic pH or oxidative microenvironment	Disease-responsive H_2_S release	Release depends on sufficiently acidic/oxidative microenvironment, which is heterogeneous within lesions	Unpredictable release in early-stage or mixed-microenvironment lesions	Inflammation, cancer, oxidative injury	[114]
Nanoparticles and MOFs	NO, CO, H_2_S, or multi-gas	Encapsulation, adsorption, or stimulus-responsive release	High loading capacity, tunable release, improved targeting	Complex synthesis/scale-up; potential immunogenicity or long-term accumulation	Regulatory pathway for inorganic/metal–organic nanomaterials less established than small molecules	Cancer, inflammation, cardiovascular disease	[115,116,117]
Polymeric micelles, liposomes, and hydrogels	NO, CO, H_2_S	Sustained, local, or injectable delivery	Improved stability, local retention, biocompatibility	Batch-to-batch variability; limited long-term storage stability	Manufacturing reproducibility and GMP scale-up remain major hurdles	Regenerative medicine, wound healing, local inflammation	[118,119,120]
Cell membrane-coated or exosome-based systems	NO, CO, H_2_S	Biomimetic membrane camouflage (e.g., macrophage-, cardiomyocyte-, or platelet-derived) enabling immune evasion and passive/active disease-site targeting	Prolonged circulation, reduced immune clearance, inherent disease-relevant targeting (e.g., inflamed or ischemic tissue) via source-cell surface markers	Complex, low-yield production; donor-source and batch variability; challenging drug/gas loading efficiency	High manufacturing cost, lack of standardized production protocols, and unresolved long-term biodistribution/immunogenicity data limit clinical scalability	Atherosclerosis-targeted therapy, myocardial ischemia–reperfusion injury, inflammation-targeted delivery	[115,121]

**Table 6:** **Future Perspectives and Clinical Translation of Precision Gas** **Medicine.**

Challenge	Current Limitation	Emerging Technology/Strategy	Potential Clinical Application	Future Direction	Key References
Biomarker Identification	Lack of reliable biomarkers reflecting local gasotransmitter activity and signaling status	Multi-omics profiling, spatial transcriptomics, metabolomics, redox proteomics, liquid biopsy	Early disease detection, patient stratification, therapeutic monitoring	Personalized biomarker-guided gasotherapy	[124]
Quantification of Endogenous Gases	Difficulty measuring NO, CO, and H_2_S concentrations in specific tissues in real time	Fluorescent probes, electrochemical biosensors, molecular imaging, wearable sensing devices	Dynamic monitoring of disease progression and treatment response	Real-time precision gas monitoring systems	[123,125]
Dose Optimization	Narrow therapeutic window and concentration-dependent toxicity	Programmable release systems, AI-assisted dose prediction, pharmacokinetic modeling	Individualized dosing regimens	Adaptive precision gas medicine	[103,107,111,113,114]
Tissue-Specific Delivery	Rapid diffusion and lack of target specificity	MOFs, liposomes, polymeric micelles, exosomes, cell membrane-coated nanoparticles	Organ-specific delivery to heart, brain, liver, kidney, and tumors	Precision tissue-targeted therapy	[115,116,117,118,119,120,121,122]
Multi-Gas Integration	Most current approaches focus on a single gasotransmitter	Multi-gas co-delivery platforms and stimuli-responsive nanomaterials	Reconstitution of physiological gasotransmitter networks	Programmable gasotransmitter network therapy	[115,116,117,118,119,120,121,122]
Disease Heterogeneity	Variable responses among patients and disease stages	Molecular classification, machine learning, systems biology approaches	Patient stratification and treatment selection	Precision medicine-based intervention	[124,126]
Monitoring Cellular Fate	Lack of biomarkers reflecting ferroptosis, autophagy, and mitophagy status	Ferroptosis-associated lipidomics, circulating miRNAs, mitochondrial biomarkers	Monitoring therapeutic efficacy and disease progression	Cell-fate-guided therapy optimization	[93,94,95,96,97,98,99,100,101]
Clinical Translation	Limited clinical trials and regulatory frameworks	GMP-compliant gas donors, scalable nanomedicine manufacturing, regulatory harmonization	Translation into cardiovascular, neurodegenerative, inflammatory, metabolic, and cancer therapies	Evidence-based gasotransmitter therapeutics	[118,119,120,121,122,124]
Artificial Intelligence Integration	Massive and complex gasotransmitter-related datasets	AI-assisted multi-omics integration, digital twin modeling, predictive analytics	Predictive diagnosis and therapeutic planning	Digital twin-guided precision gasotherapy	[127]
Long-Term Safety	Incomplete understanding of chronic exposure effects	Longitudinal cohort studies and real-world evidence platforms	Improved safety assessment and risk prediction	Personalized long-term treatment management	[118,119,120,121,122,124]

## Data Availability

No new data were created or analyzed in this study. Data sharing does not apply to this article.

## Abbreviations

3-MST	3-Mercaptopyruvate Sulfurtransferase
5-LOX	5-Lipoxygenase
ACSL4	Acyl-CoA Synthetase Long-Chain Family Member 4
AI	Artificial Intelligence
Akt	Protein Kinase B
AMPK	AMP-Activated Protein Kinase
ARE	Antioxidant Response Element
ATG	Autophagy-Related Protein
ATP	Adenosine Triphosphate
BH4	Tetrahydrobiopterin
CBS	Cystathionine β-Synthase
CORMs	Carbon Monoxide-Releasing Molecules
CO	Carbon Monoxide
COX IV	Cytochrome c Oxidase Subunit IV
CSE	Cystathionine γ-Lyase
DAMPs	Damage-Associated Molecular Patterns
DMT1	Divalent Metal Transporter 1
eNOS	Endothelial Nitric Oxide Synthase
EPR	Enhanced Permeability and Retention
ER	Endoplasmic Reticulum
FAD	Flavin Adenine Dinucleotide
FMN	Flavin Mononucleotide
Fe^2+^	Ferrous Iron
GMP	Good Manufacturing Practice
GPX4	Glutathione Peroxidase 4
GSH	Glutathione
H_2_S	Hydrogen Sulfide
HIF-1α	Hypoxia-Inducible Factor-1 Alpha
HNO	Nitroxyl
HO	Heme Oxygenase
HO-1	Heme Oxygenase-1
HO-2	Heme Oxygenase-2
IRI	Ischemia–Reperfusion Injury
KATP	ATP-Sensitive Potassium Channel
Keap1	Kelch-Like ECH-Associated Protein 1
LC3	Microtubule-Associated Protein 1 Light Chain 3
LPCAT3	Lysophosphatidylcholine Acyltransferase 3
MAPK	Mitogen-Activated Protein Kinase
MOF	Metal–Organic Framework
mPTP	Mitochondrial Permeability Transition Pore
mTOR	Mechanistic Target of Rapamycin
mTORC1	Mechanistic Target of Rapamycin Complex 1
NF-κB	Nuclear Factor Kappa B
NO	Nitric Oxide
NOS	Nitric Oxide Synthase
nNOS	Neuronal Nitric Oxide Synthase
NORMs	Nitric Oxide-Releasing Molecules
NOX	NADPH Oxidase
Nrf2	Nuclear Factor Erythroid 2-Related Factor 2
Parkin	E3 Ubiquitin Ligase Parkin
Persulfidation	Protein Persulfidation (Sulfhydration)
PI3K	Phosphoinositide 3-Kinase
PINK1	PTEN-Induced Kinase 1
PKG	Protein Kinase G
PLGA	Poly(Lactic-co-Glycolic Acid)
PTMs	Post-Translational Modifications
PUFA-PL	Polyunsaturated Fatty Acid Phospholipids
ROS	Reactive Oxygen Species
RNS	Reactive Nitrogen Species
S-Nitrosylation	Protein S-Nitrosylation
sGC	Soluble Guanylate Cyclase
SSNO^−^	Nitrosopersulfide
System Xc^−^	Cystine/Glutamate Antiporter
TfR1	Transferrin Receptor 1
TPP^+^	Triphenylphosphonium
ULK1	Unc-51-Like Kinase 1
VSMC	Vascular Smooth Muscle Cell
