# Correction: Lee, T.-S. Intracellular Crosstalk of the Gasotransmitter Trio (NO, CO, H_2_S) in Cardiovascular Health and Disease: From Molecular Signaling to Precision Gas Medicine. *Int. J. Mol. Sci.* 2026, *27*, 6248

**DOI:** 10.3390/ijms27198666

**Published:** 2026-09-28

**Authors:** Tzong-Shyuan Lee

**Affiliations:** Graduate Institute and Department of Physiology, College of Medicine, National Taiwan University, Taipei 10051, Taiwan; ntutslee@ntu.edu.tw

**Text Correction**

The reference [26] has been miscited and has to be replaced from the original publication [1]. Also, Three new references are added to the reference list as entries [125–127], respectively.

In Table 3 (“Bidirectional Regulation Among CBS, CSE, HO, and NOS”) and in the accompanying text, the citation supporting the statement that NO suppresses cystathionine γ-lyase (CSE)-dependent H_2_S-generating activity via S-nitrosation of Cys229 was incorrectly given as reference [26], which describes an unrelated finding (H_2_S-enhancing NO signaling).

A correction has been made to Section 3.2.2 (“Reciprocal Inhibition Under Pathological/High Concentrations”) and Table 3 (NO → CSE row), final paragraph of Section 3.2.2:

Reference [26] as cited for the NO → CSE interaction is replaced with: Fernandes, D.G.F.; Nunes, J.; Tomé, C.S.; Zuhra, K.; Costa, J.M.F.; Antunes, A.M.M.; Giuffrè, A.; Vicente, J.B. Human Cystathionine γ-Lyase Is Inhibited by s-Nitrosation: A New Crosstalk Mechanism between NO and H_2_S. *Antioxidants* **2021**, *10*, 1391. https://doi.org/10.3390/antiox10091391. This reference is added to the reference list as a new entry and is cited in place of reference [26] in Table 3 and in the corresponding sentence of Section 3.2.2, which describes NO-mediated S-nitrosation of Cys229 suppressing CSE-dependent H_2_S production.

In Table 4 (“Regulatory roles of NO, CO, and H_2_S in ferroptosis, autophagy, and mitophagy”), reference [66] (Antelmann, H.; Helmann, J.D. Thiol-based redox switches and gene regulation) was cited within the citation range [9,22,66,88,89] supporting statements on CO’s effects on ferroptosis and autophagy/mitophagy. Reference [66] is a general review of thiol-based redox switches and does not specifically support these CO-related statements. It has been removed from this citation range only; the corrected citation range in Table 4 (CO row) is [9,22,88,89]. Reference [66] remains in the reference list unchanged, as it continues to support an unrelated statement in Section 2.2.3; no renumbering of the reference list is required for this item.

In Table 6 (“Future Perspectives and Clinical Translation of Precision Gas Medicine”), several rows cited overly broad, overlapping reference ranges that did not accurately reflect the content of each row. Reference [123] (Cortese-Krott et al., on nitrosopersulfide chemistry) had been included in the citation ranges for the “Tissue-Specific Delivery,” “Multi-Gas Integration,” “Clinical Translation,” and “Long-Term Safety” rows, none of which concern SSNO^−^ chemistry; it has been removed from these four rows, which are corrected to [115–122], [115–122], [118–122,124], and [118–122,124], respectively. The “Biomarker Identification” row is corrected from [121–124] to [124]. The “Quantification of Endogenous Gases” row is corrected from [123,124] to [123,125], with a new reference added: Lu, C.; Zhang, C.; Zhang, X.-B.; Song, G. In vivo imaging of gasotransmitters. *Nat. Rev. Bioeng*. **2026**, *4*, 653–674. https://doi.org/10.1038/s44222-026-00417-7. The “Disease Heterogeneity” row is corrected from [121–124] to [124,126], with a new reference added: Wang, Y.; Aivalioti, E.; Stamatelopoulos, K.; Zervas, G.; Mortensen, M.B.; Zeller, M.; Liberale, L.; Vece, D.D.; Schweiger, V.; Camici, G.G.; et al. Machine learning in cardiovascular risk assessment: Towards a precision medicine approach. *Eur. J. Clin. Investig*. **2025**, *55*, e70017. https://doi.org/10.1111/eci.70017. The “Artificial Intelligence Integration” row is corrected from [121–124] to [127], with a new reference added: Sadée, C.; Testa, S.; Barba, T.; Hartmann, K.; Schuessler, M.; Thieme, A.; Church, G.M.; Okoye, I.; Hernandez-Boussard, T.; Hood, L.; et al. Medical digital twins: Enabling precision medicine and medical artificial intelligence. *Lancet Digit. Health* **2025**, *7*, 100864, https://doi.org/10.1016/j.landig.2025.02.004.

In addition, the following references in the reference list have been corrected for bibliographic accuracy (e.g., journal abbreviation, punctuation, and DOI corrections); these corrections do not affect the scientific content or conclusions of the article:

[17] Kashfi, K.; Olson, K.R. Biology and therapeutic potential of hydrogen sulfide and hydrogen sulfide-releasing chimeras. *Biochem. Pharmacol.* **2013**, *85*, 689–703. https://doi.org/10.1016/j.bcp.2012.10.019.

[26] Fernandes, D.G.F.; Nunes, J.; Tomé, C.S.; Zuhra, K.; Costa, J.M.F.; Antunes, A.M.M.; Giuffrè, A.; Vicente, J.B. Human Cystathionine γ-Lyase Is Inhibited by s-Nitrosation: A New Crosstalk Mechanism between NO and H_2_S. *Antioxidants* **2021**, *10*, 1391. https://doi.org/10.3390/antiox10091391.

[29] Cooper, C.E.; Brown, G.C. The inhibition of mitochondrial cytochrome oxidase by the gases carbon monoxide, nitric oxide, hydrogen cyanide and hydrogen sulfide: Chemical mechanism and physiological significance. *J. Bioenerg. Biomembr.* **2008**, *40*, 533–539. https://doi.org/10.1007/s10863-008-9166-6.

[46] Krylatov, A.; Maslov, L.; Tsibulnikov, S.Y.; Voronkov, N.; Boshchenko, A.; Downey, J.; Mentzer, R. The role of reactive oxygen species, kinases, hydrogen sulfide, and nitric oxide in the regulation of autophagy and their impact on ischemia and reperfusion injury in the heart. *Curr. Cardiol. Rev.* **2021**, *17*, e230421186874. https://doi.org/10.2174/1573403X16666201014142446.

[47] Sun, X.; Wu, S.; Mao, C.; Qu, Y.; Xu, Z.; Xie, Y.; Jiang, D.; Song, Y. Therapeutic potential of hydrogen sulfide in ischemia and reperfusion injury. *Biomolecules* **2024**, *14*, 740. https://doi.org/10.3390/biom14070740.

[48] Chai, J.; Zhu, J.; Tian, Y.; Yang, K.; Luan, J.; Wang, Y. Carbon monoxide therapy: A promising strategy for cancer. *J. Mater. Chem. B* **2023**, *11*, 1849–1865. https://doi.org/10.1039/D2TB02599J.

[50] Zhu, Y.; Chen, R.; Ren, H.; Li, X.; Zheng, X. Controlled delivery of gasotransmitters for cardiovascular therapy: Molecular mechanisms, engineered platforms, and translational perspectives. *Int. J. Nanomed.* **2026**, *21*, 612298. https://doi.org/10.2147/IJN.S612298.

[52] Förstermann, U.; Münzel, T. Endothelial nitric oxide synthase in vascular disease: From marvel to menace. *Circulation* **2006**, *113*, 1708–1714. https://doi.org/10.1161/CIRCULATIONAHA.105.602532.

[60] Nakamura, T.; Lipton, S.A. Emerging role of protein–protein transnitrosylation in cell signaling pathways. *Antioxid. Redox Signal*. **2013**, *18*, 239–249. https://doi.org/10.1089/ars.2012.4703.

[70] Cortese-Krott, M.M.; Fernandez, B.O.; Kelm, M.; Butler, A.R.; Feelisch, M. On the chemical biology of the nitrite/sulfide interaction. *Nitric Oxide* **2015**, *46*, 14–24. https://doi.org/10.1016/j.niox.2014.12.009.

[71] Irvine, J.C.; Ritchie, R.H.; Favaloro, J.L.; Andrews, K.L.; Widdop, R.E.; Kemp-Harper, B.K. Nitroxyl (HNO): The Cinderella of the nitric oxide story. *Trends Pharmacol. Sci.* **2008**, *29*, 601–608. https://doi.org/10.1016/j.tips.2008.08.005.

[72] Kemp-Harper, B.K. Nitroxyl (HNO): A novel redox signaling molecule. *Antioxid. Redox Signal.* **2011**, *14*, 1609–1613. https://doi.org/10.1089/ars.2011.3937.

[76] Altaany, Z.; Ju, Y.; Yang, G.; Wang, R. The coordination of S-sulfhydration, S-nitrosylation, and phosphorylation of endothelial nitric oxide synthase by hydrogen sulfide. *Sci. Signal*. **2014**, *7*, ra87. https://doi.org/10.1126/scisignal.2005478.

[81] Bucci, M.; Papapetropoulos, A.; Vellecco, V.; Zhou, Z.; Zaid, A.; Giannogonas, P.; Cantalupo, A.; Dhayade, S.; Karalis, K.P.; Wang, R.; et al. cGMP-dependent protein kinase contributes to hydrogen sulfide-stimulated vasorelaxation. *PLoS ONE* **2012**, *7*, e53319. https://doi.org/10.1371/journal.pone.0053319.

[82] Gross, G.J.; Peart, J.N. KATP channels and myocardial preconditioning: An update. *Am. J. Physiol. Heart Circ. Physiol*. **2003**, *285*, H921–H930. https://doi.org/10.1152/ajpheart.00421.2003.

[83] Mustafa, A.K.; Sikka, G.; Gazi, S.K.; Steppan, J.; Jung, S.M.; Bhunia, A.K.; Barodka, V.M.; Gazi, F.K.; Barrow, R.K.; Wang, R.; et al. Hydrogen sulfide as endothelium-derived hyperpolarizing factor sulfhydrates potassium channels. *Circ. Res.* **2011**, *109*, 1259–1268. https://doi.org/10.1161/CIRCRESAHA.111.240242.

[87] Cairo, G.; Recalcati, S.; Mantovani, A.; Locati, M. Iron trafficking and metabolism in macrophages: Contribution to the polarized phenotype. *Trends Immunol.* **2011**, *32*, 241–247. https://doi.org/10.1016/j.it.2011.03.007.

[89] Kwon, M.Y.; Park, E.; Lee, S.J.; Chung, S.W. Heme oxygenase-1 accelerates erastin-induced ferroptotic cell death. *Oncotarget* **2015**, *6*, 24393–24403. https://doi.org/10.18632/oncotarget.5162.

[93] Pickles, S.; Vigié, P.; Youle, R.J. Mitophagy and quality control mechanisms in mitochondrial maintenance. *Curr. Biol*. **2018**, *28*, R170–R185. https://doi.org/10.1016/j.cub.2018.01.004.

[96] Lee, S.J.; Ryter, S.W.; Xu, J.F.; Nakahira, K.; Kim, H.P.; Choi, A.M.K.; Kim, Y.S. Carbon monoxide activates autophagy via mitochondrial reactive oxygen species formation. *Am. J. Respir. Cell Mol. Biol.* **2011**, *45*, 867–873. https://doi.org/10.1165/rcmb.2010-0352OC.

[99] Zhou, H.; Wang, S.; Hu, S.; Chen, Y.; Ren, J. ER-mitochondria microdomains in cardiac ischemia-reperfusion injury: A fresh perspective. *Front. Physiol.* **2018**, *9*, 755. https://doi.org/10.3389/fphys.2018.00755.

[103] Nguyen, K.T.; Wu, Z.; Huang, T.; Tay, C.Y. Molecular design and medicinal applications of nano-nitric oxide delivery systems. *Curr. Med. Chem.* **2018**, *25*, 1420–1432. https://doi.org/10.2174/0929867324666170407141222.

[123] Cortese-Krott, M.M.; Fernandez, B.O.; Santos, J.L.T.; Mergia, E.; Grman, M.; Nagy, P.; Kelm, M.; Butler, A.R.; Feelisch, M. Nitrosopersulfide (SSNO^−^) accounts for sustained NO bioactivity of S-nitrosothiols following reaction with sulfide. *Redox Biol*. **2014**, *2*, 234–244. https://doi.org/10.1016/j.redox.2013.12.031.

**References**

With this correction, three new references [125–127] and an additional replacement reference (cited in place of former reference [26]) have been added to the reference list; the order of some subsequent references has been adjusted accordingly. The authors state that the scientific conclusions are unaffected. This correction was approved by the Academic Editor. The original publication has also been updated.
